# Dopamine, Erectile Function and Male Sexual Behavior from the Past to the Present: A Review

**DOI:** 10.3390/brainsci12070826

**Published:** 2022-06-24

**Authors:** Maria Rosaria Melis, Fabrizio Sanna, Antonio Argiolas

**Affiliations:** Department of Biomedical Sciences, Section of Neurosciences and Clinical Pharmacology, University of Cagliari, 09042 Monserrato, Italy; fabrizio.sanna@unica.it (F.S.); argiolas@unica.it (A.A.)

**Keywords:** dopamine, dopaminergic systems, sexual motivation, penile erection, sexual behavior, RHA/RLA rats, NEHR/NELR rats, DAT-KO rats

## Abstract

Early and recent studies show that dopamine through its neuronal systems and receptor subtypes plays different roles in the control of male sexual behavior. These studies show that (i) the mesolimbic/mesocortical dopaminergic system plays a key role in the preparatory phase of sexual behavior, e.g., in sexual arousal, motivation and reward, whereas the nigrostriatal system controls the sensory-motor coordination necessary for copulation, (ii) the incertohypothalamic system is involved in the consummatory aspects of sexual behavior (penile erection and copulation), but evidence for its role in sexual motivation is also available, (iii) the pro-sexual effects of dopamine occur in concert with neural systems interconnecting the hypothalamus and preoptic area with the spinal cord, ventral tegmental area and other limbic brain areas and (iv) D_2_ and D_4_ receptors play a major role in the pro-sexual effects of dopamine. Despite some controversy, increases or decreases, respectively, of brain dopamine activity induced by drugs or that occur physiologically, usually improves or worsens, respectively, sexual activity. These findings suggest that an altered central dopaminergic tone plays a role in mental pathologies characterized by aberrant sexual behavior, and that pro-erectile D_4_ receptor agonists may be considered a new strategy for the treatment of erectile dysfunction in men.

## 1. Introduction

Dopamine (3,4-dihydroxyphenylethylamine) is a catecholamine formed in two enzymatic steps, the first involving tyrosine hydroxylase (TH), which converts the amino acid L-tyrosine (4-hydroxyphenylalanine) to 3,4-dihydroxyphenylalanine (L-DOPA), and the second by L-DOPA-decarboxylase, which converts L-DOPA to dopamine (see [1,2]). These enzymatic pathways were originally characterized in the adrenal medulla, where dopamine was considered the precursor of noradrenaline, which is formed by dopamine β-hydroxylase that adds a hydroxyl (OH) group in the first position of the dopamine ethyl chain, and of adrenaline, which is formed from noradrenaline by phenylethanolamine N-methyl transferase that adds a methyl (CH_3_) group in the amine group of noradrenaline [1,2,3,4,5,6,7]. The idea that dopamine was only the precursor of noradrenaline changed in 1957, when the studies of Arvid Carlsson (Nobel Prize winner for Physiology and Medicine in 2000) and his co-workers revealed that dopamine is also a central neurotransmitter [8,9]. Together with the studies that allowed the mapping and identification of catecholaminergic and serotoninergic pathways in the central nervous system by using the Falck-Hillarp fluorescent method first, and immunohistochemical methods later [10,11], and the identification of selective receptors for each monoamine across the brain [12,13,14,15,16,17,18,19,20], the most important brain dopaminergic systems (e.g., nigrostriatal, mesolimbic-mesocortical, incertohypothalamic and tuberoinfundibular systems) were described and found thereafter to be deeply involved in different central functions (see [21]). Among these are motor and posture control (lesions of nigrostriatal dopaminergic neurons produce Parkinson disease symptoms) [22,23,24], motivation and reward processes (alterations of the activity of mesolimbic/mesocortical dopaminergic neurons interfere with motivation and rewarding processes and are involved in drug dependence and addiction) (see [25,26]), thought, ideation and reasoning (alterations in the activity of mesolimbic/mesocortical dopaminergic system take place in psychosis and schizophrenia) [9,12,16,27,28] and hypothalamic control of prolactin release from the adenohypophysis (tuberoinfundibular neurons release dopamine from the median eminence in the hypophysial blood portal system, allowing it to reach the adenohypophysis, where the catecholamine acts to inhibit prolactin release) [29,30,31] (Table 1).

Pioneering studies in the 1970s also revealed that dopamine was involved in penile erection and sexual behavior. Such studies were conducted mainly in laboratory rats by altering pharmacologically central dopamine and/or serotonin content and/or activity, revealed the classic dichotomy dopamine-serotonin, with dopamine facilitating and serotonin inhibiting male sexual behavior, respectively [32,33,34,35,36,37,38,39,40], whereas both neurotransmitters were found to be inhibitory to female sexual behavior [41,42,43,44,45]. However, no evidence was provided by these earlier studies on the dopaminergic system(s) involved in the facilitatory role of dopamine and of the inhibitory role of serotonin in penile erection and male sexual behavior. This was ascertained for dopamine in 1986 when it was discovered that dopamine agonists, known for their ability to induce penile erection by activating dopamine D2 receptors when given systemically in male rats, induce penile erection when microinjected into the paraventricular nucleus of the hypothalamus (PVN) [46] and facilitate copulatory behavior when microinjected into the medial preoptic area [47], respectively, revealing for the first time that the activation of the incertohypothalamic dopaminergic system, whose neurons originate in the A13 and A14 catecholaminergic cell groups of the hypothalamus [11], arborize extensively and reach several hypothalamic nuclei, is involved in the facilitatory effect of dopamine on penile erection and sexual activity. It is now clear that other dopaminergic systems, other than the incertohypothalamic one, are also involved in sexual behavior and, in particular, the mesolimbic and mesocortical systems, whose neurons have cell bodies in the ventral tegmental area and their neuronal endings in the nucleus accumbens and medial prefrontal cortex, and that play a main role in the motivational and rewarding processes, including those related to sexual activity. Moreover, the identification of numerous dopamine receptors belonging to the D1 (D_1_ and D_5_) and D2 (D_2_, D_3_ and D_4_) receptor families, has led in recent years to the synthesis of molecules that act selectively as agonists or antagonists of these dopamine receptor subtypes. This has reopened the research aimed at defining the exact role of the different dopamine receptor subtypes, mainly of the D2 family (D_2_, D_3_ and D_4_), present in the areas of the brain controlling the different aspects of sexual behavior (PVN, medial preoptic area, ventral tegmental area, nucleus accumbens, medial prefrontal cortex, and bed nucleus of the stria terminalis), from sexual motivation to erectile function and copulatory behavior (Table 2, Table 3 and Table 4).

This work reviews early and recent studies on the role of dopamine with its central neuronal systems and different receptor subtypes in the various aspects of male sexual behavior, from penile erection ex copula (in the absence of a receptive female or in the presence of an inaccessible receptive female) to copulatory behavior with a receptive female, in the species most studied so far, e.g., rats, but also other animal species including men. The aim is to show first to what extent the results of the recent studies confirm or confute the old theories suggesting that dopamine facilitates sexual behavior, and second, to present an updated description of the lastest discoveries and identify new possible strategies for the therapy of erectile dysfunction and/or other related sexual dysfunctions by modifying the activity of dopamine at the central level. The studies selected for review were chosen from Pubmed and Google Scholar medlines done with search terms such as dopamine; dopamine agonists and antagonists; erectile function; and male sexual behavior in different animal species, from rat to man only, on the basis of the presence of experiments aimed at studying the sexual role of dopamine in Section 7. No method was used to validate and/or summarize the evidence for and against the facilitatory role of dopamine in penile erection and sexual behavior given by the selected studies.

## 2. Dopamine and Erectile Function

**Figure 1:**
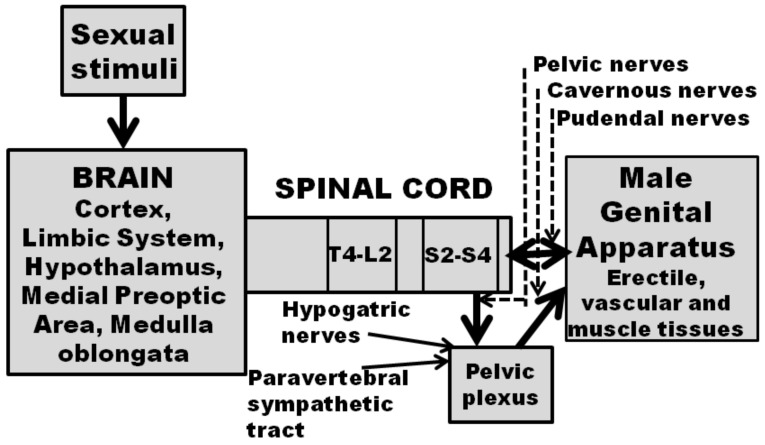
A simplified representation of the neural pathways that control penile erection and male sexual behavior. When sexual (visual, auditory, olfactory, tactile and even imaginative in man) stimuli reach the brain’s high centers, these activate neural pathways (to date still unknown), which lead to penile erection, the key event for copulation/sexual intercourse. These travel from the brain, mainly from the hypothalamus and its nuclei (i.e., paraventricular nucleus) and medial preoptic area, along the medulla oblongata and the spinal cord, to the genital apparatus. The latter is innervated by pudendal nerves that originate from the sacral (S2–S4) spinal tract and in which run the primary afferent sensory from and motor pathways to the penis, and by cavernous nerves in which run the primary efferent sympathetic and parasympathetic pathways originating in the pelvic plexuses. These receive neural inputs from pelvic nerves originating in the sacral (S2–S4) spinal tract, from hypogastric nerves that originate in the thoracic-lumbar (T4–L2) spinal tract and post-gangliar fibers that originate from the paravertebral sympathetic ganglia of the thoracic-lumbar tract of the spinal cord (T11–L2) (for references see [82,100,101,102]).

Penile erection is produced by a complex neural central and peripheral interaction which induces muscle and vascular changes in the erectile tissues of the male genital apparatus (cavernous corpora, corpus spongiosum, bulbocavernous and ischiocavernous muscles and other perineal muscles). This interaction is also complicated by humoral and endocrine influences, mediated mainly by testosterone and its metabolites, which occur at the central and peripheral levels (see [82,83,84,85]). This sexual response can take place during sexual activity and in other contexts, for instance after manipulation of the genitalia, during sleep or erotic fantasies in men, or in male rats in the presence of an inaccessible receptive female rat (non-contact erections), or after treatment with different drugs (e.g., dopamine agonists, serotonin agonists, nitric oxide donors, phosphodiesterase inhibitors, soluble guanylate cyclase activators, RhoA-Rho kinase inhibitors, etc.) and neuropeptides [e.g., adrenocorticotropin (ACTH)-melanonocyte stimulating hormone (α-MSH)-related peptides, oxytocin, hexarelin analogues, VGF related peptides and others], which act centrally or peripherally [83,86,87,88,89,90,91,92,93,94,95,96,97,98,99,100,101,102]. Depending on the context in which this sexual response occurs, it is commonly accepted that different central and peripheral neural and/or humoral endocrine mechanisms may participate in the regulation of penile erections, usually in a very complex manner [99,100] (see Figure 1 for a simplified representation of central and peripheral neural pathways controlling penile erection and sexual behavior). The participation of dopamine in erectile function was discovered in the 1980s when dopamine D2-like receptor agonists were found able to induce penile erection in experimental ex copula settings, e.g., in male rats kept alone in a cage in the absence of a receptive female rat.

### 2.1. Dopamine D2-Like Receptor Agonists Given Systemically Induce Penile Erection in Rats, Mice, Monkeys and also in Man

As recalled above, in the 1980s, numerous mixed D1/D2 or D2 dopamine receptor agonists administered systemically at low, but not at high doses were found capable of inducing penile erection in ex copula conditions, first in male rats put alone in a cage in the absence of a receptive female [103,104,105,106,107] and later also in other animals, including monkeys and man [108,109,110,111,112,113]. The pro-erectile effect of D2-like receptor agonists was usually abolished by pretreatment with low doses of D2 receptor antagonists able to cross the blood–brain barrier (i.e., haloperidol, pimozide, L-sulpiride,), but not by domperidone, a selective D2 receptor antagonist that does not cross the blood–brain barrier [114,115], suggesting that dopamine facilitates erectile function by acting on D2 receptors in the central nervous system (for a review see [108]). The inability of D2 receptor agonists given at high doses to induce penile erection was explained with the occurrence of marked stereotyped behaviors that mask the appearance of penile erection [104,107], although an inhibitory action of the drugs at the level of the spinal cord level cannot be ruled out [116,117]. The stimulatory effect of dopamine agonists on penile erection is abolished by castration and reversed by testosterone or estradiol benzoate plus 5α-dihydrotestosterone replacement therapy, but not by estradiol benzoate or 5α-dihydrotestosterone administered alone [118]. These findings are in line with the permissive role of testosterone and/or its neural metabolites in the induction of penile erection by dopamine agonists and of erectile function in general. The above studies were soon followed by studies showing that dopamine receptor agonists induce penile erection when microinjected into the PVN of male rats [46] and modify penile reflexes induced by retraction of the penile sheath of rats maintained in a supine position (reflex erections) when administered in the medial preoptic area [119], providing the first evidence for a role of the incertohypothalamic dopaminergic system in the control of this sexual function, whose main role in male sexual activity does not need to be stressed.

### 2.2. Dopamine D2-Like Receptor Agonists Induce Penile Erection in Male Rats by Activating PVN Oxytocinergic Neurons Projecting to Extrahypothalamic Brain Areas and the Spinal Cord

**Figure 2:**
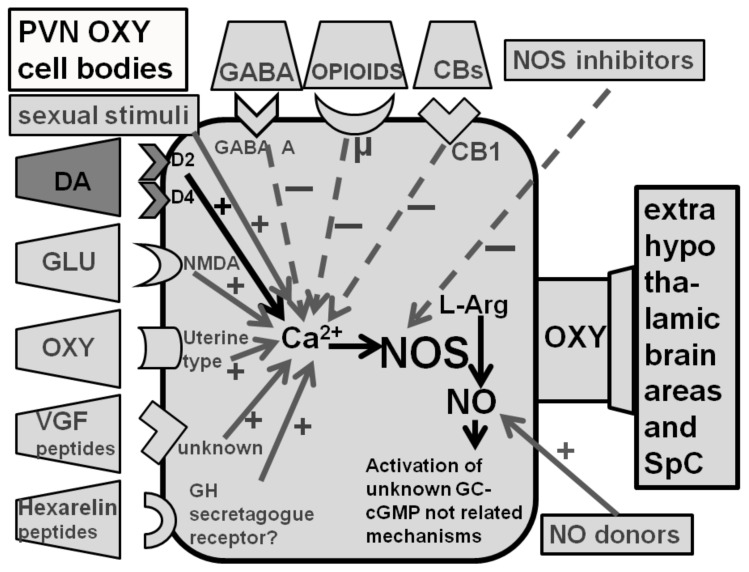
Dopamine facilitates penile erection by activating central oxytocinergic neurons in the PVN. The main mechanism by which dopamine and dopamine D2-like receptor agonists (D_2_ and D_4_ receptor subtypes) facilitate penile erection and copulatory activity is the activation of oxytocinergic neurons that originate in the paraventricular nucleus of the hypothalamus (PVN) and project to the spinal cord (SpC) and extra-hypothalamic brain areas. Briefly, dopamine, (DA) by acting on D_2_ and D_4_ receptors located in the cell bodies of these oxytocinergic neurons, increases Ca^2+^ ions influx in these cell bodies, causing in turn the activation of nitric oxide synthase (NOS), a Ca^2+^ calmodulin-dependent enzyme present in oxytocinergic cell bodies which converts the amino acid L-arginine (L-Arg) to nitric oxide (NO). NO in turn activates oxytocinergic neurons that release oxytocin in the spinal cord and in extra-hypothalamic brain areas to induce penile erection and facilitate sexual behavior by a mechanism that does not involve the guanylate cyclase-cyclic guanosine monophosphate (GC-cGMP) pathway. These oxytocinergic neurons facilitate penile erection and copulatory behavior when activated by dopamine (pathway shown in black) and by other substances, i.e., oxytocin, NMDA, hexarelin and VGF peptides and by drugs that increase PVN NO content (NO donors) by the blockade of PVN CB1 receptors (which are not located on oxytocinergic cell bodies but when blocked stimulate the activity of the latter ones by increasing glutamatergic neurotransmission in the PVN) and by physiological sexual stimuli (i.e., pheromones) (pathways indicated in gray). Conversely, when the activity of these oxytocinergic neurons is inhibited, for instance by GABA, opioid peptides/opiate drugs or drugs that inhibit NO synthase activity, the spontaneous (e.g., physiologically activated) or drug/neuropeptide-stimulated erectile function and copulatory activity is reduced (pathways also indicated in gray) (for references see [82,100,101,102]). This figure is an adapted version of a figure published by the authors in [102].

The studies reviewed above did not provide any information on the location of dopamine D2 receptors whose activation led to penile erection. This was discovered in 1986, when the mixed D1/D2 agonist apomorphine and the D2 agonist quinpirole (LY 171,555), but not the D1 agonist SKF 38,393 (2,3,4,5-tetrahydro-7,8-dihydroxy-1-phenyl-1H-3-benzazepine), were found to be able to induce penile erection in a dose-dependent manner when injected in the PVN, but not in other brain areas [46]. This hypothalamic nucleus contains synapses of dopaminergic neurons belonging to the A14 group of the incertohypothalamic system [120,121] that impinge, among others, on the cell bodies of oxytocinergic neurons [120,121], including the parvocellular oxytocinergic neurons, which project to extrahypothalamic brain areas, the medulla oblongata and the spinal cord (Figure 2), (see [100,101,122] and references therein). Several lines of experimental evidence show that dopamine and dopamine receptor agonists induce penile erection by acting on dopamine D2-like receptors present in PVN oxytocinergic cell bodies. Accordingly, apomorphine-induced penile erection is reduced dose-dependently either by the administration of nonapeptide oxytocin receptor antagonists, d(CH_2_)_5_-Tyr(Me)^2^-Orn^8^-vasotocin, Pen^1^,Phe(Me)^2^,Thr^4^,Orn^8^-oxytocin and d(CH_2_)_5_-Tyr(Me)^2^-Arg^8^-vasopressin given intracerebroventricularly, but not in the PVN, with a potency parallel to their rank order in blocking oxytocinergic receptors [123] or by PVN electrolytic lesions [124], which destroy central oxytocinergic projections and cause a total elimination of central oxytocin content [125,126]. Apparently, stimulation of D2 receptors increases Ca^2+^ influx in the oxytocinergic cell bodies. Accordingly, penile erection induced by apomorphine administered into the PVN of male rats is abolished by the prior administration of ω-conotoxin, a potent antagonist of N-type Ca^2+^ channels [127,128], and by pertussis toxin, which catalyzes the irreversible ADP-ribosylation of several G proteins, including the Go protein coupled to voltage-dependent calcium channels [129,130]. These findings suggest that PVN D2 receptors mediating penile erection localized in the parvocellular oxytocinergic cell bodies are coupled to ω-conotoxin-sensitive N-type Ca^2+^ channels [128] through a pertussis toxin sensitive G protein [130]. In line with this hypothesis, the activation of dopamine receptors of the D2 type present in the cell bodies of oxytocinergic neurons that mediate penile erection increases the production of nitric oxide in the PVN. In neurons, nitric oxide is formed by neuronal nitric oxide synthase, a member of a family of Ca^2+^-calmodulin dependent enzymes that convert the amino acid L-arginine in citrulline and nitric oxide. This molecule was added in 1990 to the neurotransmitters/neuromodulators that control smooth muscle relaxation in all vascular tissues across the body (see [131]), and is considered at the penile level to be the main mediator of the relaxation of cavernous corpora, the key event of penile erection at the local level [132,133,134]. It is pertinent to recall that the PVN is one of the hypothalamic nuclei richest in neuronal nitric oxide synthase, which is present among others in the cell bodies of oxytocinergic neurons mediating penile erection ([135] and references therein). Thus, the stimulation of dopamine receptors of the D2 type in the PVN by dopamine agonists increases Ca^2+^ influx in the oxytocinergic cell bodies, leading in turn to the activation of nitric oxide synthase, thus increasing nitric oxide content in the PVN. Several lines of experimental evidence show that newly formed nitric oxide activates oxytocinergic neurons projecting to extrahypothalamic brain areas and to the spinal cord, mediating penile erection by acting intracellularly with a still unknown mechanism not related to the activation of guanylate cyclase, which converts GTP to cGMP and that is one of the best known targets of nitric oxide (see [101] and references therein). Irrespective of the mechanism by means of which nitric oxide activates oxytocinergic neurons in the PVN, that D2 agonists activate oxytocinergic neurons originating in the PVN and projecting to extra-hypothalamic brain areas is confirmed by experiments showing that: (i) apomorphine given at doses that induce penile erection increases the oxytocin concentration in the hippocampus as well as in plasma [136] and (ii) apomorphine-induced penile erection is markedly impaired by lesions of the medial septum, which decreases hippocampal oxytocin by about 50% due to the lesion of the paraventricular–hippocampal oxytocinergic projection [137]. Together with the ability of oxytocin to induce penile erection when also administered to the hippocampus [138], these findings provided convincing evidence that dopamine agonists facilitate erectile function by increasing central oxytocin neurotransmission in extrahypothalamic brain areas and in the spinal cord by acting in the PVN. Numerous studies have also shown that similar mechanisms occur when oxytocinergic neurons are activated by other neurotransmitters and agonists of their receptors and other compounds (excitatory amino acids, VGF derived peptides, hexarelin analogue peptides, nitric oxide donors, etc.), which facilitate penile erection by activating oxytocinergic neurotransmission in the PVN, like dopamine ([100,101] and references therein). Conversely, many of the inhibitory or facilitatory influences of other neurotransmitters on apomorphine- and other dopamine agonist-induced penile erection as those exerted by GABA agonists (i.e., muscimol), opioid peptides and/or drugs that act on opioid receptors (morphine, naloxone) may be mediated by modifications of central oxytocinergic neurotransmission ([100,101,139] and references therein). Perhaps more relevant for this review and as it will be discussed below, a significant increase in extracellular dopamine is found in the PVN dialysate when penile erection occurs in physiological contexts, as when sexually potent male rats put in the presence of an inaccessible receptive female, show non- contact erections (pheromone-induced penile erections) and during copulation as well when in copula penile erections take place [140]. This suggests that incertohypothalamic dopamine activity increases in the PVN in those physiological contexts that lead to the induction of penile erection, in line with an important role of the incertohypothalamic dopaminergic system in penile erection and sexual activity. This finding resembles the increase in extracellular dopamine that takes place in the nucleus accumbens during copulatory activity in male rats [141,142,143] and confirms that different dopaminergic systems are activated during sexual activity, in line with a specific role of these dopaminergic systems in the two main phases of sexual behavior (see Section 5, Section 5.1 and Section 5.2).

### 2.3. Dopamine Agonists of the D1 and D2 Type Receptors Exert an Opposite Role on Penile Reflexes in Male Rats at the Level of the Medial Preoptic Area and the PVN

Dopamine agonists also modify the number of penile reflexes induced by retraction of the penile sheath in restrained male rats placed in a tube in a supine position (reflex erections). Indeed, as seen with penile erection, apomorphine given subcutaneously at low doses increases by 70% and at high doses decreases by 40% the number of these spinally-mediated penile reflexes [116,119,144,145]. Similar findings are also found measuring the latency to the appearance of the reflex, with low, but not high doses that decrease the time until the first reflex [116,119,145]. This effect is antagonized by haloperidol, but not by domperidone, which does not cross the blood–brain barrier [145]. In this test, at all doses, apomorphine facilitated seminal emission, independently of the occurrence of penile erection [145]. Similar findings are found with apomorphine injected into the medial preoptic area [48,119,146,147] or into the PVN [119,147,148]. It is likely that the PVN and the medial preoptic area cooperate in the control of these responses, since they are in reciprocal contact and both contain incertohypothalamic dopaminergic nerve endings [116,120,121]. Conversely, the dopamine antagonist α-flupenthixol decreases penile reflexes and impairs copulation when injected in the medial preoptic area [147]. Although these results are similar to those seen by studying the effect of dopamine agonists on penile erection in freely moving rats, thus suggesting a facilitatory role of central D2 receptors in the induction of penile reflexes, this interpretation is complicated by the findings obtained with the D2 agonist LY 163,502, which unexpectedly decreases penile reflexes by 70% when given systemically [144] or in the medial preoptic area [48,146] despite its ability to facilitate seminal emission in both cases [146] and with the D1 antagonist SCH 23,390 [R(+)-7-chloro-8-hydroxy-3-methyl-1-phenyl-2,3,4,5-tetrahydro-1H-3-benzazepine hydrochloride], injected in the medial preoptic area, which decreases penile reflexes, as found with LY 163,502, whereas the full D1 agonist THP (dihydroxyphenyl-tetrahydrothienopyridine) increases them, suggesting that D1 and D2 receptors play an opposite role in the control of this response [50]. Whatever the role of D1 and D2 receptors may be in the regulation of spinal reflexes and seminal emission, these findings support the main role of dopamine in the medial preoptic area and thus of the incertohypothalamic dopaminergic system in the regulation of spinal penile reflexes and ejaculatory threshold. 

### 2.4. Dopamine D_2_ and D_4_, but Not D_3_ Receptor Subtypes, Mediate Dopamine Agonist-Induced Penile Erection by Activating PVN Oxytocinergic Neurons That Project to Extrahypothalamic Brain Areas and the Spinal Cord

In the 1990s, it was definitively ascertained by molecular biology studies that dopamine D1 and D2 receptors are two families of receptors, which include the first D_1_ and D_5_ receptors subtypes and the second D_2_ (long and short splice variants, D_2L_ and D_2S_), D_3_ and D_4_ receptor subtypes (for a review on dopamine receptors subtypes see [13,14,15,16]). This led to the synthesis and characterization of numerous molecules that act selectively on the above receptor subtypes at least in intact cultured cells in which the different dopamine receptor subtypes have been inserted by molecular biology techniques and in in vitro binding studies in membranes obtained from cultured cells expressing the cloned dopamine receptors and from brain tissues as well (Table 3 and Table 4). A few years later, immunohistochemistry experiments revealed that dopamine D_2_, D_3_ and D_4_ receptors are localized in the cell bodies of parvocellular oxytocinergic neurons in the PVN [149,150]. Microinjection studies with mixed dopamine receptor agonists (e.g., the mixed D1/D2 agonist apomorphine and the mixed D_3_/D_2_ agonist pramipexole) and a few of these new selective agonists and antagonists of the D_2_, D_3_ and D_4_ receptor subtypes given systemically or into the PVN revealed that (i) penile erection induced by mixed dopamine agonists injected into the PVN is mediated mainly by the stimulation of D_2_, but not the D_3_ receptor subtype [74,75] and (ii) the selective stimulation of D_4_ receptors also induces penile erection (but not yawning), but this receptor subtype apparently does not play a role in penile erection induced by mixed dopamine agonists apomorphine and pramipexole [58,59,60,61,78,79,80] (Table 5), although some controversy exists on these two points [51,52]. Accordingly, apomorphine- and pramipexole-induced penile erection is antagonized by selective D_2_, but not by D_3_ or D_4_ dopamine receptor antagonists, and D_4_ dopamine receptor antagonists abolish penile erection induced by D_4_ dopamine receptor agonists, but exert only minor effects on apomorphine- [79,80] and no effect at all on pramipexole-induced erectile response [74,75,76,77,78,79,80]. In combination with intracerebral microdialysis experiments, the above studies confirmed that penile erection induced by D_2_ receptor agonists given to the PVN takes place concomitantly with an increase in the PVN nitric oxide production, confirming that dopamine in this hypothalamic nucleus facilitates erectile function by activating nitric oxide synthase located in the cell bodies of oxytocinergic neurons that project to extrahypothalamic brain areas and the spinal cord mediating penile erection (Figure 2). Paraventricular D_4_ receptor agonist-induced penile erection also occurred together with an increase in paraventricular nitric oxide production in line with the hypothesis that D_4_ receptors also facilitate erectile function by activating oxytocinergic neurons mediating penile erection (Figure 2). Accordingly, penile erection induced by D_2_ and D_4_ receptor agonists administered into the PVN were both significantly reduced either by the prior administration into the PVN of neuronal nitric oxide synthase inhibitors and by oxytocin receptor antagonists injected into the lateral ventricles, but not into the PVN [75,79,80] (Table 6).

Against the main role of D_2_ receptors in the erectile response induced by mixed dopamine agonists apomorphine and pramipexole are the results of a paper showing that pramipexole-induced penile erection is antagonized by the dopamine D_3_ receptor antagonist SB277011A, which led the authors of the study to suggest that D_3_ rather than D_2_ receptors mediate pramipexole-induced penile erection [51]. However, another study confirms that D*_2_* and not D_3_ receptors are those that mediate the pramipexole erectile response, and suggests that the involvement of D_3_ receptors supported by the previous study may be due to the dose of antagonist used, as that dose was high enough to block both D_2_ and D_3_ receptors rather than D_3_ receptors only [52]. These discrepancies are due to the fact that of the numerous molecules synthesized and identified for a selective effect on these dopaminergic receptors in vitro, only a few interact with a selectivity high enough to discriminate among the D_2_, D_3_ and D_4_ receptor subtypes when expressed in cultured transfected cells (Table 4). In particular, although many of these molecules act mainly as rather selective antagonists of the D_2_, D_3_ and D_4_ receptors, it has been difficult and it is still unknown how to obtain agonists that act with high selectivity on the D_2_ and the D_3_ receptors, respectively (that is, compounds with a different affinity for these two receptors have been identified, but this different affinity is not high enough to allow a selective activation of only one of the two receptors without affecting also the other one). D_2_ and D_3_ agonists able to cross the blood—brain barrier and are more selective than those available so far have to be tested for their effect on penile erection to clarify definitively this point. Irrespective of the involvement of D_3_ receptors or not in apomorphine- and/or pramipexole-induced penile erection, unexpectedly both these studies failed to show the proerectile effect of D_4_ agonists, thus raising some doubt on the proerectile effect of D_4_ agonists in male rats reported above, a finding that is against the results of the studies reviewed above in which the proerectile effect of several selective D_4_ agonists was clearly demonstrated [58,59,60,61,74,75,78,79,80]. The proerectile role of D_4_ receptors was discovered in 2004 during the screening of new molecules aimed at characterizing the dopamine receptor subtypes involved in the facilitatory effect of mixed dopamine receptor agonists on penile erection. ABT-724, a selective dopamine D_4_ agonist, was found to be able to induce penile erection when given systemically to, and into the lateral ventricles of male rats, with potency similar to that of apomorphine [58]. In agreement with the results of these studies, other D_4_ agonists, i.e., CP226,269, PD 168,077, PIP-3EA, A-412,997, FAUC 3019 and FAUC 389 (Table 4), were found capable of inducing penile erection [59,79]. Some of these compounds were found able to induce penile erection when given not only systemically and into the lateral ventricles, but also into the PVN, apparently by activating oxytocinergic neurons mediating penile erection by a mechanism similar to that of apomorphine and D_2_-like receptor agonists, e.g., by increasing oxytocinergic neurotransmission [59,60,61,74,75,78,79,150,151]. Accordingly, similar to apomorphine, PD 168,077 and ABT 724 injected into the PVN at doses that induce penile erection increased nitric oxide content in the PVN dialysate obtained by intracerebral microdialysis, and this effect was antagonized by nitric oxide synthase inhibitors injected into the PVN prior the injection of the D_4_ agonists at doses that reduce the D_4_ agonist-induced erectile response [79,151]. Penile erection induced by PD 168,077 and ABT 724 was also abolished by the potent blocker of voltage dependent Ca^2+^ channels ω-conotoxin GVIA given to the PVN prior the D_4_ agonists, and by the selective oxytocin receptor antagonist d(CH_2_)_5_-Tyr(Me)^2^-Orn^8^-vasotocin, given intracerebroventricularly but not into the PVN (Table 6) [79,80,151]. Finally, similar to apomorphine, PD 168,077 given to the PVN at doses that facilitate penile erection also increased extracellular dopamine concentration in the dialysate obtained by intracerebral microdialysis from the nucleus accumbens shell [80]. Since L-745,870, a selective D_4_ receptor antagonist, was found capable of abolishing PD 168,077- and ABT 724-, but not pramipexole- and apomorphine-induced penile erection and associated responses (e.g., increased nitric oxide production in the PVN, extracellular dopamine increase in the nucleus accumbens dialysate) [74,75,78,79,80,151], and functional D_4_ receptors are found in the PVN together with D_2_ and D_3_ receptor subtypes, all being located in the cell bodies of oxytocinergic neurons [149,150,152], these studies led to suggest that D_4_ receptor agonists induce penile erection by acting in the PVN on D_4_ receptors whose stimulation activates oxytocinergic neurons controlling penile erection (but not yawning) [58,75,79,151]. Finally, since some of these D_4_ receptor agonists, (e.g., ABT 724) unlike apomorphine, were found ineffective in inducing emesis in the ferret, a classic animal model of emesis in mammals [153,154], this reopened the research on dopamine receptor agonists able to induce penile erection devoid of the collateral emetic effects of classic mixed dopamine receptor agonists.

## 3. Dopamine Released in the Nucleus Accumbens Also Facilitates Penile Erection

The studies reviewed above provide evidence for a role of incertohypothalamic dopaminergic neurons in the regulation of penile erection at level of the PVN and the medial preoptic area. Several lines of evidence also support a proerectile role of the mesolimbic and mesocortical dopaminergic systems, whose neurons originate in the ventral tegmental area and project the first to the nucleus accumbens and the second to the medial prefrontal cortex, respectively. Accordingly, extracellular dopamine increases in the nucleus accumbens dialysate obtained from male rats injected with oxytocin into the ventral tegmental area at a dose that induces penile erection by activating mesolimbic/mesocortical dopaminergic neurons [100,155,156,157,158,159,160] (Table 7). The release of dopamine in the nucleus accumbens is required for the proerectile effect of oxytocin, since this behavioral response is antagonized by the dopamine receptors blockade in the nucleus accumbens by the potent dopamine receptor antagonist haloperidol, given to the nucleus accumbens before oxytocin, at doses that do not induce gross behavioral changes [155]. Oxytocin also induces penile erection when given to the ventral subiculum of the hippocampus or to the posteromedial cortical nucleus of the amygdala [158] (Table 7). Apparently, oxytocin injected into these two brain areas activates directly or indirectly glutamic acid activity in the ventral tegmental area [159,160]. This increase in glutamic acid neurotransmission in the ventral tegmental area causes in turn the activation of mesolimbic/mesocortical dopaminergic neurons, and an extracellular dopamine increase in the nucleus accumbens and medial prefrontal cortex, leading to penile erection as reported above for oxytocin given directly into the ventral tegmental area. Accordingly, a blockade of excitatory amino acid receptors of the NMDA subtype in the ventral tegmental area by dizolcipine [(+) MK-801] abolishes the proerectile response of oxytocin given to the ventral subiculum or the amygdala (Table 7) [159,160]. 

Extracellular dopamine increases in the dialysate obtained from the nucleus accumbens of male rats when also injected into the PVN with the mixed D1/D2 dopamine agonist apomorphine or with the selective D_4_ agonist PD 168,077 given at a dose that induces penile erection by activating oxytocinergic neurons that project from the PVN to extra-hypothalamic brain areas, including the ventral tegmental area, the ventral subiculum of the hippocampus and the posteromedial cortical nucleus of the amygdala [80]. Additionally, in this case, the erectile responses of apomorphine and PD 168,077 were the first antagonized by the blockade of dopamine D_2_ receptors and the second of the D_4_ subtype present in the PVN and both by the blockade of oxytocin receptors in the ventral tegmental area by d(CH_2_)_5_Tyr(Me)^2^-Orn^8^-vasotocin (Table 7) [80], as found for the erectile response of oxytocin given to the ventral tegmental area [155,157]. As discussed below (Section 7), in view of the key role of mesolimbic dopaminergic neurons in sexual motivation and reward, these findings have contributed to the hypothesis that incertohypothalamic and mesolimbic dopaminergic neurons participate with central oxytocinergic neurons that originate in the PVN and project to extrahypothalamic brain areas and glutamatergic neurons to a neural circuit that regulates the control of both the consummatory and anticipatory components of sexual behavior [98,102,151,161]. 

## 4. Dopamine Released in the Bed Nucleus of the Stria Terminalis Also Facilitates Penile Erection

In comparison with mesolimbic dopaminergic neurons that when activated by oxytocin injected into the ventral tegmental area at doses that induce penile erection, increase dopamine release in the nucleus accumbens, dopaminergic neurons that project from the ventral tegmental area to the bed nucleus of the stria terminalis are also involved in the proerectile response of oxytocin given to the bed nucleus of the stria terminalis, the last brain area discovered until now where oxytocin is able to induce penile erection (and yawning) [162,163]. In fact, neuropharmacological and microinjection studies first [162] and microdialysis and immunohistochemical experiments later [163] show that oxytocin given to the bed nucleus of the stria terminalis induces penile erection (and yawning) by activating both glutamatergic (and nitrergic) and dopaminergic neurotransmission in this brain nucleus. Accordingly, this complex structure contains the nerve terminals of (i) glutamatergic neurons that originate in the ventral subiculum of the hippocampus and/or the amygdala (see [164,165] and references therein), and impinge on glutamatergic neurons that contain nitric oxide synthase and project to the PVN and/or the medial preoptic area, the ventral tegmental area, the ventral subiculum and/or the amygdala, activating here the neural pathways mediating penile erection; and (ii) of dopaminergic neurons that originate in the ventral tegmental area ([164,165] and references therein) and impinge on the same glutamatergic nerve terminals in which are located oxytocinergic receptors whose activation causes the release of glutamic acid. Apparently, dopamine released by oxytocin acts on D1, but not D2 receptors that stimulate the same glutamatergic neurons rich in nitric oxide synthase that project to the PVN and/or the medial preoptic area, activating the neural pathways mediating penile erection present in these brain areas. In line with this hypothesis, the blockade of dopamine D1 receptors and excitatory amino acid receptors in the bed nucleus of the stria terminalis by the D1 receptor antagonist SCH 23,390, or by the amino acid receptors of the NMDA and AMPA receptor subtype antagonists (+) MK-801 and CNQX (6-cyano-7-nitroquinoxaline-2,3-dione), respectively, antagonizes penile erection induced by oxytocin injected into the bed nucleus [162]. Moreover, double labelling immunohistochemistry shows also that oxytocin-labelled neuronal elements are in close proximity to tyrosine hydroxylase-labelled (dopaminergic) neurons or nitric oxide synthase-labelled cell bodies surrounded by intense vesicular glutamate transporter1-stained (glutamatergic) synapses in the bed nucleus sections in which oxytocin injections induce the above responses [163]. As discussed below (Section 7), these results confirm the involvement of the bed nucleus of the stria terminalis in a complex neural circuit controlling both the consummatory (erectile function and copulatory performance) and the anticipatory phases of sexual behavior (sexual arousal and motivation) through dopaminergic, glutamatergic and oxytocinergic pathways that interconnect often reciprocally in many of the brain areas recalled above [100,101,102,161] (Section 8). 

## 5. Dopamine and Sexual Behavior

Sexual behavior plays a fundamental role in the reproduction of all living animals, from insects to mammals, including humans. In mammals, it is generally accepted that it occurs in two main phases, anticipatory and consummatory, and different quantifiable parameters have been identified in each phase and in males and females [166,167,168,169,170,171,172,173]. These studies were done mainly in rats because of their availability, the well-characterized sequence of copulatory behavior and its parameters in the male (for a detailed description of the male rat copulatory behavior see [87,173,174,175,176]), and of proceptive and receptive (lordotic) behavior in the female (see [177,178]), although data on other animal species are also available [179,180,181,182,183,184,185]. Penile erection, mounts and intromissions, seminal emission and ejaculation characterize the consummatory phase of male sexual response, whereas vaginal lubrication, clitoris erection and orgasm are typical of female sexual response. These consummatory responses are preceded by an anticipatory/appetitive phase, which includes motivation towards and searching of a partner for copulation [87,175]. Additionally, in humans, the sexual response is organized in distinct and sequential phases, which usually include (but not always) sexual desire followed by sexual arousal and orgasm, including ejaculation in males with a partner available for sexual intercourse, although an integration of these phases is likely to exist (see [186,187,188]). Briefly, when sexual (visual, auditory, olfactory, tactile and in humans even imaginative) stimuli reach the central nervous system, this activates neural pathways that drive sexual information from the higher brain centres through the spinal cord and the autonomous nervous system to the genital apparatus leading to penile erection in males and vaginal lubrication/clitoral erection in females in order to make sexual intercourse feasible ([82,83,87,133,134,189,190] and references therein) (Figure 1). It is known that many neurotransmitters and neuropeptides play a role at the central and peripheral level in the two phases of sexual behavior. Of neurotransmitters, dopamine has been and is still certainly one of the most studied. As recalled in the Introduction (Section 1) and above, in 1970s, pioneering studies showed that drugs that modify dopamine and serotonin content or their function at the central level have dramatic effects on the sexual behavior of rats and other laboratory animals, mainly rats because of their availability, the well-known sequence of copulatory behavior and its parameters in this animal species [174,175] and of proceptive and receptive (lordotic) behavior in the female rat [177,178]. These studies led to propose that dopamine and serotonin have an opposite role in sexual activity, the first facilitating and the second inhibiting sexual behavior in males [32,33,35,36,37,38,39,40,191], whereas both compounds were found to be inhibitory in females [41,42,43,44,192]. Since then, the function of dopamine in sexual behavior has been investigated using numerous procedures, from lesion studies with selective neurotoxins, microinjections of agonists and antagonists into given brain areas and finally with several new techniques for studying neurotransmitter function in vivo, i.e., intracerebral microdialysis, differential pulse voltammetry and chronoamperometry, either in the anticipatory and/or consummatory phase of sexual behavior. Many of the early studies done since 1965 up to 1995 have been extensively and excellently reviewed by different authors (see [69,193] and references therein). Since then, our knowledge on dopamine neurotransmission has been greatly increased by the molecular characterization of different dopamine receptors of the D1 (D_1_ and D_5_) and D2 (D_2_, D_3_ and D_4_) families, with their specific splice variants. This has initiated the search for molecules that act as selective agonists or antagonists of the different dopamine receptor subtypes, in order to define their involvement in human mental illness, i.e., psychosis and schizophrenia (see [13,14,15,16]). The availability of these new agonists and antagonists of the different dopamine receptors (in particular of the D2 family) (Table 3 and Table 4) also reopened the research for identifying the role of these receptor subtypes in the control of sexual behavior. Moreover, other techniques for studying neurotransmitter function in vivo have become available, such as genetically modified mice and rats, e.g., animals knocked out for key enzymes synthesizing catecholamines (i.e., tyrosine hydroxylase and DOPA decarboxylase), proteins controlling their reuptake (i.e., dopamine transporter, DAT) and/or their metabolism (monoamine-oxidases, MAOs, and catechol-O-methyl-transferase, COMT) and perhaps even more importantly, for the different dopamine receptor subtypes recalled above. However, only a few studies are found in the literature, which have analyzed sexual behavior in these genetically modified mice and rats compared to their wild-type control counterparts. These studies usually provide support to the facilitatory role of dopamine in sexual behavior.

### 5.1. Dopamine and Male Sexual Behavior 

The first evidence for a facilitatory role of catecholaminergic systems in male copulatory behavior was provided by Soulairac and Soulairac in 1957 [32], who tested the effect of amphetamine, which releases catecholamines from dopaminergic and noradrenergic endings of central and peripheral neurons [194] in the male rat. Accordingly, amphetamine was found able to increase the number of ejaculations and to reduce the post ejaculatory interval, the frequency of intromissions and the ejaculation latency, all results which suggest a facilitatory effect of catecholamines in the copulatory behavior of male rats. This was confirmed by Tagliamonte, Gessa and their coworkers, who showed that in sluggish rats (e.g., rats that show low basal levels of sexual activity) L-DOPA, the dopamine precursor, administered with a peripheral DOPA decarboxylase inhibitor, but not alone, increased the percentage of animals reaching ejaculation by 50% [35,36], leading to suggest that the concentration of catecholamines in the brain must rise above a critical level to produce an “aphrodisiac” effect. The major involvement of dopamine versus noradrenaline in facilitating copulatory behavior was obtained by studies showing that low doses of apomorphine, a classical mixed dopamine D1/D2 receptor agonist, increased the number of rats showing mounts, intromissions and reaching ejaculation [35,36,195]. In particular, apomorphine at low doses decreased significantly mount latency and the number of mounts and intromissions for reaching ejaculation, parameters considered an index of facilitation of sexual arousal and of ejaculatory threshold, respectively. Conversely, apomorphine at high doses that induces marked interfering stereotyped behavior decreases rather than improve copulatory behavior [196]. However, the dopaminergic mechanism of the stimulatory effect of apomorphine on copulatory behavior was definitively proven by the ability of the selective dopamine receptor antagonist haloperidol to abolish apomorphine sexual effects when given at low doses. Haloperidol was also found able to impair copulatory behavior of sexually potent male rats, e.g., it increased mount and intromission latencies and decreased mount, intromission and ejaculation frequencies, effects that were much more evident when the drug was used at high doses [172,197,198]. These findings resemble the decrease in libido and sexual desire that occur in male patients under neuroleptic treatment for mental illness [199,200]. Results similar to those obtained with apomorphine and haloperidol were soon also observed with other dopamine receptor agonists, i.e., N-n-propyl-norapomorphine, bromocriptine, pergolide, lisuride, RDS-127 or by treatments which potentiate dopamine activity, i.e., clorgyline, a MAO-B inhibitor [40,104,201,202,203,204,205] and other dopamine receptor antagonists, such as cis-flupentixol, sulpiride and pimozide [172] and have also been repeatedly confirmed by recent studies [200]. However, it is important to recall that some of the dopamine agonists cited above, i.e., pergolide, lisuride and RDS-127, are also potent agonists of the serotoninergic 5HT_1A_ receptors, whose stimulation facilitates sexual behavior by impairing serotoninergic neurotransmission [206,207,208,209,210]. The facilitatory effect of these drugs on sexual behavior might thus reflect the concomitant activation and inhibition of the dopaminergic and serotoninergic neurotransmission, respectively, rather than the activation of dopamine neurotransmission alone. Irrespective of the exact mechanism by which pergolide, lisuride and RDS-127 facilitate male rat sexual behavior, apomorphine- and dopamine receptor agonist-induced facilitation of sexual behavior in rats is testosterone-dependent, as it is abolished by castration (which eliminates circulating testosterone) and restored by testosterone or its metabolites estradiol and 5α-dihydroxy-testosterone replacement [118], in line with the permissive role of this sexual hormone on erectile function and sexual behavior in males. 

### 5.2. Dopamine Receptors and Male Sexual Behavior 

Many of the earlier studies cited above were done when it was still unknown that dopamine acts on two main types of receptors, the D1 and the D2 receptors. This was discovered in 1979 [12,211,212], and it was made clear that either apomorphine or haloperidol act on both dopamine D1 and D2 receptors, although with different efficacies [12], complicating the understanding of the role of these two kind of receptors on male sexual behavior. Since then, different selective dopamine D1 and D2 receptor agonists and antagonists have been tested on copulatory behavior in male rats. These studies have shown that rather selective dopamine D2 agonists, such as quinpirole [45] and quinerolane [213], induce effects similar to those of apomorphine on several parameters of copulatory behavior when given systemically, e.g., low doses improve and high doses impair male rat copulatory behavior [214], in line with the hypothesis that dopamine D2 receptors are those that play a major role in the facilitatory effect of mixed dopamine D1/D2 agonists on male sexual behavior. However, the involvement of D1 receptors cannot be completely ruled out, due to the results of a few studies showing that the D1 agonist SKF 38,393 also can influence male rat copulatory performance at least in some experimental conditions. In fact, although in sexually experienced male rats with free access to the sexually receptive female rat, SKF 38,393 was found almost ineffective in altering copulatory parameters in spite of its ability to decrease the number of intromissions required to reach ejaculation when given either intraperitoneally [71] or subcutaneously [72], and to increase mounts number, ejaculation latency and post ejaculatory intervals [73] (e.g., findings that suggest an inhibitory role of D1 receptors on male sexual behavior). In sexually exhausted male rats, the drug was found to be able to improve sexual behavior, as inferred by the increase in the percentage of sexually exhausted rats recovering copulation after SKF 38,393 treatment, and by the increase in mounts number and the decrease in ejaculation latency, as found with apomorphine [73]. Since SKF 38,393 was also found able to increase the permanence of male rats after copulation in the compartment of an instrumental apparatus in which male rats have been trained to access in order to copulate with a sexually receptive female rat [72], these findings suggest that D1 and D2 receptors play distinct roles in the expression of male rat sexual behavior depending on the motivational state of the animals [73]. In spite of the above uncertainty about the role of D1 receptors on male sexual behavior, as discussed above for apomorphine, the facilitation of sexual behavior by dopamine agonists was explained by the activation of post-synaptic D2 receptors, whereas the inhibition was ascribed mainly to the masking of sexual behavior by behavioral effects such as stereotyped sniffing, biting, gnawing and hypermotility. Likewise, L-sulpiride, a selective dopamine D2 receptor antagonist [12], and SCH 23,390, a selective dopamine D1 receptor antagonist [215], given systemically were found to be both able to increase mount latency more than threefold, but while SCH 23,390 induced effects very similar to those found with mixed D1/D2 antagonists, i.e., haloperidol and pimozide (e.g., increased mount and intromission latencies and decreased mount, intromission and ejaculation frequencies) [172,198], L-sulpiride failed to modify sexual behavior once the copulatory sequence was initiated [172,198,216]. Similar effects on sexual behavior were also reported for other typical and atypical neuroleptic drugs, i.e., metoclopramide and clozapine, although with some differences [172,198]. Most importantly, the dopamine antagonist domperidone, which does not cross the blood–brain barrier [114,115], was found unable to alter copulatory behavior [217] or to reduce the facilitatory effect of dopamine receptor agonists [217], confirming that these drugs act on dopamine receptors localized in the brain and not in the periphery to influence male rat sexual behavior. Some discrepancy apart, from the studies reviewed above, it appeared convincing that the D2 receptors were those playing the major role in the copulatory behavior of male rats, although a modulatory role of D1 receptors could be not ruled out as discussed above [71,72,73], at least in some brain areas (e.g., the medial preoptic area) [49]. The potency of dopamine receptor agonists in inducing penile erection not only in male rats but also in men [109] and in facilitating male rat sexual behavior [38,195], also led to testing the clinical use of apomorphine with a preparation to be administered sublingually in patients suffering for erectile dysfunction. However, the success of this formulation made commercially available was scarce, especially when compared to that of phosphodiesterase type V inhibitors, possibly due to unwanted side effects of this and other dopamine agonists, such as nausea and vomiting, secondary to the stimulation of dopamine receptors located in the chemo-trigger zone outside the blood–brain barrier and which are incompatible with sexual intercourse in men (see [83] and references therein).

As already recalled in Section 2 and Section 2.1, in the 1990s, molecular biology studies led to the discovery that dopamine D1 and D2 receptors are two families of receptors, made first by D_1_ and D_5_ receptors subtypes and second by D_2_ (long and short splice variants, D_2L_ and D_2S_), D_3_ and D_4_ receptor subtypes (see [13,14,15,16] and references therein). This discovery was soon followed by the synthesis and characterization of numerous molecules acting selectively as agonists or antagonists on the above receptor subtypes in intact cultured cells in which the different dopamine receptor subtypes have been inserted by molecular biology techniques and in in vitro binding studies in membranes obtained from both cultured cells expressing the cloned dopamine receptor subtypes and brain tissues as well (see Table 3 and Table 4). The availability of these selective dopamine agonists and antagonists has produced not only the studies reviewed above that have identified the main role of dopamine D_2_ receptors in the erectile response of mixed dopamine receptor agonists and the ability of D_4_ agonists to induce penile erection, but also a few studies aimed at characterizing the role of these dopamine receptor subtypes on male sexual behavior and to the identification of molecules able to facilitate sexual behavior as found with mixed dopamine receptor agonists, but devoid of the unwanted effects of the latter, e.g., compounds that facilitate penile erection and sexual behavior unable to induce nausea and vomiting. In fact, as discussed in Section 8, these compounds might represent a valid strategy for the treatment of erectile dysfunction of central origin in men alternative to the use of locally acting phosphodiesterase inhibitors.

### 5.3. D_4_ Dopamine Receptors Agonists and Antagonists Improve and Impair Sexual Behavior in Male Rats: Comparison with Mixed D2-Like Dopamine Receptor Agonists and Antagonists

As recalled above (see Introduction, Section 1), classical mixed dopamine receptor agonists, such as apomorphine, pramipexole, quinpirole and quinelorane, not only facilitate penile erection in ex copula contexts (e.g., in the absence of any sexual stimuli, as the presence of a receptive female), but also facilitate sexual behavior of sexually potent male rats, as inferred by the changes induced on the sexual parameters measured in classical copulation tests with an ovariectomized receptive (estrogen + progesterone-primed) female rat, mainly a decrease in mount and intromission frequency and ejaculation latency, and an increase in ejaculation frequency (see [198] and references therein). Despite the studies quoted above, supporting a proerectile action of D_4_ receptor agonists, to our knowledge, only one detailed study is available which compares the effect of two rather selective D_4_ receptor agonists, PD 168,077 and ABT-724, given systemically, with that of apomorphine on male rat copulatory behavior [70]. Briefly, this study shows that these two tested D_4_ receptor agonists improve the copulatory behavior of sexually potent male rats, e.g., the two compounds decreased dose-dependently mount frequency and post ejaculatory interval and increased ejaculation frequency, without altering the other copulatory parameters (Table 8). 

Many of these changes resemble those induced by apomorphine, except the post ejaculatory interval, which was not modified by apomorphine, but shortened by the two D_4_ receptor agonists. On the other hand, apomorphine also altered other copulatory parameters, i.e., it decreased ejaculation latency and intromission frequency, which were not modified by PD 168,077 or ABT-724 (Table 8). In line with the facilitatory effect of PD 168,077 and ABT-724 on male rat copulatory behavior, this study also shows that L-745,870, a selective D_4_ dopamine receptor antagonist inhibited copulatory behavior of sexually potent male rats, e.g., increased intromission and ejaculation latency, mount frequency and post ejaculatory intervals, and decreased ejaculation frequency [70] (Table 9). These changes resemble those found with haloperidol, a classic dopamine receptor antagonist that blocks all D_2_ receptor subtypes, although this drug modified almost all copulatory parameters as expected, including those not altered by the D_4_ receptor antagonist, indeed, haloperidol was also very effective in increasing mount latency and in reducing intromission frequency, parameters not modified by L-745,870 (Table 9). Finally, in line with the hypothesis that PD 168,077 and ABT-724 facilitate copulatory behavior by acting mainly on D_4_ receptors, L-745,870 given before PD 168,077 or ABT-724, abolished almost completely the improving effects of both D_4_ receptor agonists on copulatory parameters, without altering or inducing only minor effects on the facilitatory sexual responses of apomorphine [70] (Table 10).

### 5.4. Differences between Mixed Dopamine D_2_-like Receptor Agonists and D_4_ Receptor Agonists on Penile Erection and Copulatory Behavior

As described in the studies reviewed above (Section 2.1 and Section 2.2), mixed dopamine D2 receptor agonists and D_4_ receptor agonists induce both penile erection and facilitate sexual behavior in male rats. These effects are apparently mediated by the same mechanism, that is both classes of drugs stimulate dopamine D2-like receptors localized in the cell bodies of PVN oxytocinergic neurons that project to extrahypothalamic brain areas and to the spinal cord ([78,79,80,155,157] and references therein). The activation of these receptors causes an increase in Ca^2+^ ions influx that leads to the activation of nitric oxide synthase, increasing nitric oxide production in the oxytocinergic neuronal cell bodies. Newly formed nitric oxide in turn activates oxytocinergic neurons facilitating erectile function and copulatory behavior [75,101,102,151,157]. However, despite the similarity of the mechanisms of dopamine mixed D_2_-like receptor agonists with those of D_4_ receptor agonists, which mediate the sexual effects of these compounds, a few differences are evident that deserve some comment. First, all together the findings reviewed above on the facilitatory effects of the two D_4_ receptor agonists, PD-168,077 and ABT-724, on male copulatory behavior suggest that these sexual effects are secondary to the selective activation of D_4_ receptors only. This does not apply to apomorphine, as this drug improves male copulatory behavior by acting on more dopamine receptor subtypes rather than on D_4_ receptors only (see below). In fact, if apomorphine sexual effects were secondary to the selective stimulation of D_4_ receptors, they should have been antagonized by L-745,870, which blocks D_4_ receptors selectively. This is not the case since L-745,870 exerted only minor effects on or does not abolish at all apomorphine effects on copulatory behavior (Table 10) [79,155,157]. In view of the failure of L-745,870 to also prevent the facilitatory effects of the other mixed dopamine D_2_/D_3_ agonist pramipexole on erectile function [74,75] (see Section 2.4), it is also likely that the above hypothesis also applies to the facilitatory effects of this mixed dopamine D_2_/D_3_ receptor agonist on copulatory behavior [77,218], although no study on the effect of L-745,870 or other D_4_ receptor antagonists on the pramipexole-induced facilitation of copulatory behavior was found in the available literature.

Second, the selective stimulation of D_4_ receptors induces penile erection and facilitates copulatory behavior with an efficacy lower than that of mixed D2-like dopamine receptor agonists, e.g., apomorphine. The reason for such difference is unknown. One possibility may be that mixed D2-like dopamine receptor agonists interact with D_4_ receptors with a higher affinity than PD 168,077 and ABT-724. Accordingly, in vitro studies show that (i) the EC50 of apomorphine (6.0 nM) is much lower than that of PD-168,077 (83 nM) and ABT-724 (281 nM) in increasing the GTPγS binding activity in CHO cells transfected with human D_4_ receptors (e.g., apomorphine activates G-protein coupled D_4_ receptors after receptor binding at doses much lower than those of PD-168,077 and ABT-724 (see [55,219]) and (ii) the EC50 of apomorphine (4.3 nM) is lower than that of PD-168,077 (5.6 nM) and of ABT-724 (12.4 nM) in increasing Ca^2+^ influx in HEK-293 cells co-transfected with rat/human D_4_ receptors and a G protein (Gαq05) (see [154] and references therein). Alternatively, PD 168,077 and ABT-724 may act as dopamine D_4_ receptor partial agonists, or the concomitant activation of different dopamine receptor subtypes by apomorphine or pramipexole may lead to a higher activation of the targets that mediate penile erection and copulation than the activation of one dopamine receptor subtype only.

Third, although haloperidol, which blocks all D_2_-like receptors, was more effective than L-745,870 in impairing copulatory behavior, the blockade of D_4_ receptors by the highest dose of L-745,870, given in the above study, showed a tendency to decrease the number of rats that show intromission and ejaculation in the first series of copulatory activity. This effect was not observed with haloperidol, which failed to affect this parameter at the doses used in this study. The tendency of L-745,870 to decrease the number of rats that become engaged in intromissions and reached ejaculation occurred even if L-745,870-treated male rats showed mounts with the receptive female. One explanation of this finding is that the selective blockade of D_4_ receptors interferes with the occurrence of penile erection in in copula conditions. In this regard, it is noteworthy that L-745,870 was also reported to be capable of reducing not only apomorphine- and pramipexole-induced ex copula penile erections episodes by about 30% [74,75,78,79], but also the percentage of rats that showed intromission when put with a receptive female for a period of 15 min when given to the lateral ventricles [150]. 

Finally, the last difference worthy of note between the mixed dopamine D2-like receptor and the selective D_4_ receptor agonists used in the studies reviewed here, e.g., PD 168,077 and ABT-724, is that the latter do not induce yawning [75]. This is at variance from apomorphine and pramipexole, which are both very effective in eliciting this behavioral response when the two drugs are tested for their ability to induce spontaneous penile erection episodes in the absence of sexual stimuli, as with those that occur in the presence of a sexually receptive female rat. This suggests that the selective stimulation of D_4_ receptors activates the neural pathways that lead to penile erection and sexual behavior, but are not those responsible for the yawning response, which are activated by the concomitant stimulation of D_2_, D_3_ and D_4_ receptors by apomorphine and pramipexole and other mixed D2-like dopamine agonists as well. Irrespective of the exact mechanisms at the basis of the differences discussed above between the erectile and copulatory effects produced by the stimulation of all D2-like receptors as that obtained with mixed dopamine agonists and with the stimulation of D_4_ receptors only, these differences reveal that dopamine may influence differentially erectile function, copulatory behavior and its two main phases and other functions as well (i.e., the yawning response) by acting selectively or in concert with its receptors in a given brain area only or in different brain areas.

## 6. Rats with a Higher Dopaminergic Tone Than Their Control Counterparts Obtained by Selective Breeding Protocols over Multiple Generations or by Genetic Mutations Show Improved Erectile Function and Copulatory Behavior

The studies reviewed so far show that dopamine has a facilitatory role in penile erection and copulatory behavior and that this is secondary to the activity of different dopaminergic neuronal systems across the brain, from the incertohypothalamic and tuberoinfundibular systems, which are involved mainly in erectile and copulatory performance, to the mesolimbic-mesocortical systems involved mainly in sexual motivation and reward processes, although a cooperative interaction exists between these systems (see Section 8). That central dopamine activity is important for erectile function and sexual behavior is also confirmed by studies done in rats in which dopamine neurotransmission has been altered by psychogenetic selections as those occurring when two groups of animals that show opposite specific behavioral traits are selected by means of repeated inbreeding or outbreeding procedures over multiple generations, which lead to the appearance of a higher dopaminergic tone in one of the two groups when compared to the other, or by genetic mutations of enzymes and/or proteins that control dopamine synthesis and content in the brain. Accordingly, the animals showing an increased dopaminergic tone should be expected to show an improved erectile function and copulatory behavior when compared to those with a lower dopaminergic tone. In line with this hypothesis are studies done in rats psychogenetically selected to show a specific behavioral trait in a given situation (e.g., in a condition of novelty or stress) or for their divergent frequency in showing a spontaneous behavior (e.g., yawning), and, to our knowledge, by one study done in knockout rats for the dopamine transporter (DAT) gene (DAT-KO rats) compared to the heterozygous (DAT-HET) and wild-type (DAT-WT) rats. 

### 6.1. Erectile Function and Sexual Behavior in Rats Selectively Bred That Show a Higher Dopaminergic Tone than Their Lower Tone Counterparts

Among psychogenetically selected rat sub-lines, the most known for having a different dopaminergic tone are the Roman high- (RHA) and low-avoidance (RLA) rats originally selected for their different avoidance response in the shuttle box (see [220,221,222]), the High (bNEHR) and Low Novelty Exploration Responders (bNELR) rats selectively bred for their different exploratory behavior in a novel environment (see [223]), and the Low and High Yawning frequency (LY and HY) rats selectively bred for their divergent frequency in showing spontaneous yawning (see [224,225]), an innate response with a function still far from being understood (see [89,226,227,228,229] and references therein). Of these three couples of psychogenetically selected rat sub-lines, the most studied for their differences in copulatory behavior and the involvement of dopamine in these differences are the RHA and RLA rat sub-lines, although a few studies on this subject done in the bNEHR and bNELR and LY and HY rats are also available.

### 6.2. RHA and RLA Rats Have a Different Central Dopaminergic Tone

RHA and RLA rats are two Wistar-derived rat lines of rats that were phenotypically selected and bred for more than 30 years in Geneva (Switzerland) for rapid acquisition (RHA) versus very scarce acquisition (RLA) of two-way active avoidance in the shuttle box [220]. Studies with these Swiss Roman rat sub-lines revealed that emotional but not learning capacities were responsible for their opposite performance in this test [220,221,230]. Indeed, RLA rats are hyperemotional and show a behavioral repertory characterized by hypomotility and freezing, whereas RHA rats show a proactive coping behavior that drives a rapid learning of the avoidance response [231]. RLA rats display either higher emotionality but also higher anxiety and reactivity to numerous stressful conditions when compared to RHA rats [232,233,234,235,236,237] (for a review see [221]). RHA rats are believed a model for the study of novelty and/or sensation seeking [234,235,236,237] because they show high impulsivity levels [238]. RHA rats show a stronger mesolimbic dopaminergic response when treated with drugs of abuse, e.g., cocaine, morphine [239] and alcohol [240] than RLA rats. The two Roman rat sub-lines also differ markedly in various behavioral traits deeply influenced by dopamine neurotransmission, for instance the stereotypies and motor responses produced by direct and indirect dopamine agonists (apomorphine, amphetamine), vulnerability to and acquisition, maintenance, extinction and reinstatement of drugs of abuse (i.e., cocaine, morphine, ethanol), and fear-related behaviors (see [235,236,240,241,242,243,244,245,246]). The results of these studies led to suggest that RHA and RLA rats differ in central dopaminergic tone, with RHA rats possessing a dopaminergic activity higher than that of RLA rats, and that such a difference may be involved in many of the behavioral divergences between the two Roman rat sub-lines described above. Among these divergences very relevant for this review are the differences in the pro-erectile (and pro-yawning) response to dopamine receptor agonists and antagonists and the different copulatory pattern shown by male RHA and RLA rats placed with a sexually receptive (ovariectomized and estradiol + progesterone-primed) female rat for the first time in the mating arena for a classical copulatory test.

### 6.3. RHA and RLA Rats, Erectile Function and Dopamine

As to the differences in the effects of dopamine receptor agonists and antagonists on penile erection (and yawning), both RHA and RLA rats show penile erection (and yawning) when treated with D_2_ or D_4_ receptor agonists, this response being antagonized by dopamine D_2_ or D_4_ receptor antagonists, respectively [81]. This picture is similar to that found in normal genetically heterogeneous rats, but with striking differences occurring between RHA and RLA rats, being RLA rats are more responsive to dopamine receptor agonists, e.g., RLA show a higher number of penile erection episodes than RHA rats [81], a finding that strongly suggests a different dopaminergic tone playing a role in the divergent behavioural responses of RHA and RLA rats. On the other hand, a more marked stereotyped behavior was found with higher doses of the mixed D1/D2 dopamine receptor agonist apomorphine in RHA versus RLA rats, which masks penile erection (and yawning) episodes in RHA rats. These findings are in line with the results of a study showing a higher reduction in motility and a higher increase of yawning episodes in RLA versus RHA rats after treatment with low doses of apomorphine, whereas RHA rats were much more sensitive than RLA rats to the stereotypies caused by apomorphine given at high doses [245].

### 6.4. RHA and RLA rats, Sexual Behavior and Dopamine

As to the differences in copulatory patterns shown by male RHA and RLA rats when put together with a sexually receptive (ovariectomized and estradiol + progesterone-primed) female rat for the first time in the mating area for a classical copulatory test, in line with a higher dopaminergic tone in RHA versus RLA rats, about 80% of RHA rats become occupied with copulation and reached ejaculation against 40% of RLA rats only [247]. The percentage of RHA rats that were occupied in the first copulatory test was also higher than that of genetically heterozygous control rats (~55%). These differences between RHA and RLA rats were still found in the second and third copulatory tests, although less evident, but tended to disappear after five tests [247]. These findings show that, once sexual experience has been acquired and the effect of novelty has disappeared, RLA rats are able to perform in sexual activity as well as RHA rats, although even after five copulatory tests, the percent of RLA rats reaching ejaculation in the first series of copulatory activity (~80%) was still lower than that of RHA rats (~100%). These results suggest that the sexual performance of RLA rats is more modest than that of RHA rats even after strong sexual experience is acquired. This was also confirmed by analysing copulatory parameters of the first series of copulatory activity along the five tests. This revealed that RLA rats have also longer latencies to start mounts and intromissions, show fewer mounts and intromissions to reach ejaculation, fewer ejaculations and a longer post ejaculatory interval than RHA rats in the copulatory tests (Table 11).

RLA rats also seem to differ from RHA rats in the levels of sexual motivation, as shown by their always longer latency to mount and intromit when compared to RHA rats in the copulatory tests (Table 11). Accordingly, as recalled above (Section 5.1) longer/shorter latencies to mount and intromit are considered indices of lower/higher levels of sexual motivation, respectively ([69,87,248,249,250] and references therein).

### 6.5. Experimental Evidence Supporting the Role of a Different Dopaminergic Tone in the Sexual Differences between RHA and RLA Rats

#### 6.5.1. Autoradiography, Binding and Behavioral Studies

Numerous lines of experimental evidence support the hypothesis that the differences in the erectile response to dopamine receptor agonists and antagonists and in the copulatory patterns between RHA and RLA rats are secondary to the different dopaminergic tone existing in the two Roman rat sub-lines. Accordingly, binding studies in brain homogenates from [251] and autoradiography studies in the two rat sub-lines [246] using radiolabeled selective D1-like and D2-like receptor antagonists revealed higher levels of D_1_ and D_3_ binding sites in the nucleus accumbens and in the medial prefrontal cortex of RHA rats and higher levels of D_3_ binding sites in the islands of Calleja of the ventral striatum of RLA rats. Reduced levels of dopamine D2 autoreceptors have also been detected by dopamine receptor binding and mRNA assays in the substantia nigra, ventral tegmental area, caudate putamen and nucleus accumbens, together with a higher dopamine release induced by amphetamine in RHA versus RLA rats [252].

Together, these results suggest that RHA rats have a nigrostriatal and mesolimbic dopaminergic tone higher than that of RLA rats due to a lower number of inhibitory presynaptic D2-like autoreceptors in the dopaminergic neuronal cell bodies of the substantia nigra and ventral tegmental area and/or to a higher number of and/or to higher affinity dopamine receptors in the nucleus accumbens, striatum and prefrontal cortex [81,246,251,252]. It is then reasonable to assume that the higher dopaminergic tone present in the limbic brain areas of RHA rats versus RLA rats is involved in the differences in copulatory behavior (discussed above) observed in the two Roman rat sub-lines (RHA rats display superior sexual performance and higher sexual motivation than RLA rats) [247,253,254,255], although other neurotransmitters (i.e., serotonin and GABA) might be also involved [230,251]. In line with this hypothesis are the results of a few behavioral studies done in sexually naive and sexually experienced RHA and RLA rats compared to those of genetically heterogeneous rats. One of these studies shows that apomorphine, a mixed dopamine D1/D2-like receptor agonist, and haloperidol, a dopamine D2-like receptor antagonist, induce differential changes on the copulatory parameters of sexually experienced RHA and RLA rats when compared to those of genetically heterogeneous control rats [253]. As expected, apomorphine improves and haloperidol impairs sexual motivation and copulatory activity in both RHA and RLA rats, but both drugs were much more efficacious in RLA than RHA rats as if RLA rats were more responsive to both the activation and the blockade of dopamine receptors than RHA rats, in line with a lower dopaminergic tone in RLA versus RHA rats [253].

#### 6.5.2. Microdialysis and Copulatory Behavior Combined Experiments

The definitive proof for the role of the higher dopaminergic tone in the limbic brain areas of RHA rats compared to RLA rats in the sexual differences found in the two Roman rat sub-lines was obtained with experiments that combined the study of copulatory behavior with intracerebral microdialysis performed in male RHA and RLA rats. These studies confirm that the higher number of non-contact penile erections that occur in the presence of an inaccessible sexually receptive female rat in RHA rats compared to RLA rats and the differences in copulatory patterns and parameters seen between RHA and RLA rats when copulation is allowed, occur in parallel with differential changes in the well-known increase in extracellular dopamine and its main metabolite 3,4-dihydroxyphenylacetic acid (DOPAC) in the dialysate from the nucleus accumbens [254] (a brain area where dopamine plays a major role in sexual motivation and reward) (see [161,172,250,256,257,258] and references therein) and from the medial prefrontal cortex (another area with dopaminergic nerve endings that may be involved in the sexual differences found between sexually naive and experienced RHA and RLA rats [255]). Briefly, in sexually naïve rats, extracellular dopamine and DOPAC increase much more in the nucleus accumbens dialysate of RHA than RLA rats. In sexually naive RHA, but not RLA rats, the increase in extracellular dopamine is already observed during the appetitive phase of sexual activity (e.g., with the inaccessible female, when male rats show non-contact erections). In sexually experienced rats, extracellular dopamine and DOPAC increase much more than in sexually naïve rats in both RHA and RLA rats, but RHA rats show a higher increase than RLA rats, as found in sexually naive RHA and RLA rats. Conversely, also in sexually experienced RHA and RLA rats, the dopamine increase in the appetitive phase is found in RHA but not RLA rats. All these differences take place even if both sexually naïve and experienced RHA and RLA rats show similar extracellular dopamine and DOPAC baseline levels during the habituation period in the nucleus accumbens dialysate, that is, before a sexually receptive female is placed in the mating cage (Table 12), in agreement with previous studies (see [240,250]).

A detailed analysis of the differential changes in extracellular dopamine of RHA and RLA rats suggest that sexual experience makes faster, rather than increases, the mesolimbic dopaminergic activity in RHA rats, while causing a general increase in the activity of mesolimbic dopaminergic neurons during copulation in RLA rats, so that their dopaminergic tone becomes functionally similar to that of sexually experienced RHA rats. Together, the higher increases in the nucleus accumbens extracellular dopamine that takes place in sexually naïve and experienced RHA rats compared to RLA rats during the appetitive and consummatory phases of sexual activity, confirm the role of a different functional tone of the mesolimbic dopaminergic system in the sexual differences found between the two Roman rat sub-lines.

Results similar to those described above are also found when measuring extracellular dopamine levels in the medial prefrontal cortex dialysate from RHA and RLA rats put in the presence of an inaccessible sexually receptive female rat and when copulatory activity is allowed as well. Even if the function of the medial prefrontal cortex in copulatory behavior is still a matter of study, extracellular dopamine and DOPAC also increase in this brain area of both male RHA and RLA rats put in the presence of an inaccessible female rat and even more significantly during copulatory activity (Table 13) [255]. These increases are seen in sexually naïve and experienced animals, but are much higher in RHA than in RLA rats, and in sexually experienced RHA and RLA rats versus their naïve counterparts, as observed in the nucleus accumbens. However, whereas the modifications in the medial prefrontal cortex extracellular dopamine release in sexually naïve and sexually experienced rats during sexual behavior takes place with similar basal dopamine levels in the medial prefrontal cortex dialysate collected before the introduction of a sexually receptive female (Table 13), the modifications in the extracellular DOPAC release take place in presence of basal DOPAC levels in RHA rats at least two-fold higher than those of RLA rats, as if dopamine function in the medial prefrontal cortex were higher in RHA than RLA rats. This raises the possibility that RHA rats have a dopaminergic tone more robust than that of RLA rats, not only in the nucleus accumbens but also in the medial prefrontal cortex [255].

To our knowledge, no study is available that is aimed at ascertaining whether changes in dopaminergic neurotransmission that resemble those reported above that take place in the mesolimbic and mesocortical dopaminergic neurons may also occur in incertohypothalamic dopaminergic neurons that innervate the medial preoptic area and the PVN of RHA and RLA rats (see Section 2.2, Section 2.3 and Section 2.4). Since these neurons are also involved in both the anticipatory (e.g., sexual motivation) and consummatory phases of sexual behavior (e.g., penile erection, ejaculation and copulatory performance) by interacting with several neuronal systems (e.g., those containing excitatory and inhibitory amino acids, nitric oxide, oxytocin and opioid peptides) [69,82,100,101,102,161,259,260,261,262,263], alterations in the density and/or affinity of dopamine receptors, mainly of D2-like receptors of the D_2_ and D_4_ subtype [74,75], and in extracellular dopamine concentrations in these brain areas as well [140,259,260,261,262,263], may have also a role in the differences in the number of non-contact erections, copulatory patterns and in the differential copulatory responses to apomorphine and haloperidol found between RHA and RLA rats [247,253,254]. Studies on dopamine neurotransmission in the medial preoptic area and the PVN of RHA and RLA rats are thus necessary to clarify this point.

### 6.6. BNEHR and BNELR Rats, Sexual Behavior and Dopamine

bNEHR and bNELR rats are two rat sub-lines originating from Sprague-Dawley rats psychogenetically selected for their extremely different response in novelty exploration, e.g., locomotion [223], with bNEHR rats vigorously exploring novel environments and bNELR rats showing limited novelty-induced activity. As found in RHA and RLA rats, these two rat sub-lines also exhibit several different behavioral traits: bNEHR rats show exaggerated aggression [264]*,* impulsivity and proclivity to addictive behaviors [265,266,267] when compared with bNELR rats, which exhibit exaggerated anxiety and immobility [268,269], depressive-like behavior and stress vulnerability [268,270,271]. Among neurobiological differences that may contribute to the high versus low novelty-seeking trait in these two rat sub-lines, differences in dopaminergic function are those that are believed to play a major role (see [265,268,272,273,274,275,276]). Accordingly, several studies have demonstrated that (i) bNEHR rats display increased basal dopaminergic activity in the nucleus accumbens, increased responses to dopaminergic drugs (e.g., psychostimulants) and lower D_2_ mRNA levels and D_2_ receptor binding sites compared to bNELR rats [267,269,272,273], (ii) bNEHR rats are more inclined to self-administer cocaine [266], whereas bNELR rats seem an useful new rodent model for the study of co-morbid depression-like behaviors and anxiety and (iii) fast-scan cyclic voltammetry studies show that bNEHR rats display a higher number of spontaneous dopamine release episodes in the nucleus accumbens and a greater reward-induced dopamine release compared to bNELR rats [265]. However, the involvement of dopamine in the above behavioral and neurochemical differences between bNEHR and bNELR rats is complicated by other studies which support the role of other neurotransmitters, e.g., serotonin ([264] and references therein) and even more noradrenaline [277]. In fact, the latter study not only confirms a different dopaminergic tone in the nucleus accumbens of the two rat sub-lines, but also shows that in this nucleus a stronger noradrenergic tone in bNEHR versus bNELR rats is present. This study also suggests that noradrenaline in the nucleus accumbens may play a main role in the higher locomotor response of bNEHR rats versus bNELR rats, since the blockade of noradrenergic receptors in this nucleus abolishes the hyperlocomotion of bNEHR rats, which was used for the selective breeding of these rat sub-lines [277]. Despite the uncertainty raised by this recent study on the unique role of dopamine in the differences between bNEHR rats versus bNELR rats, these two rat sub-lines show different patterns of copulatory behavior with a sexually receptive female rat [248], with differences in copulatory parameters that resemble those found in RHA and RLA rats [247]. Accordingly, (i) about 80% of bNEHR rats initiated copulatory behavior and reached ejaculation with a sexually receptive female for the first time, against a 50% of bNELR rats, (ii) these differences were still found in the second copulatory test but tended to disappear in the third, fourth and fifth test, and (iii) in all copulatory tests bNEHR rats display shorter mount and intromission latencies, more intromissions and shorter inter-intromission intervals than bNELR rats. These copulatory differences between bNEHR and bNELR rats occur with minor changes in the ejaculation frequency, post ejaculatory interval and copulatory efficacy [248] (Table 11). In view of the above recalled behavioral and neurochemical similarities between bNEHR and bNELR rats and RHA and RLA rats (i.e., similar behavioral and coping stiles when exposed to a novel environment or aversive stressful situations, and similar differences in dopamine function, i.e., increased basal dopaminergic activity in the nucleus accumbens, increased responses to dopaminergic drugs of bNEHR versus bNELR rats, lower D2 mRNA levels and D2 receptor binding sites in, and more proclivity to self-administer cocaine of bNEHR versus bNELR rats), it is reasonable that the differences in copulatory behaviour between bNEHR and bNELR rats are also secondary to the different central dopaminergic tone of the two rat sub-lines [266,267,269,272,273,277], as already discussed for RHA and RLA rats (see above and [253,254,255]). Unfortunately, no study was found aimed at ascertaining if differential changes in dopamine activity occur concomitantly to the different patterns of sexual behavior in bNEHR and bNELR rats in brain areas that play a role in copulatory behavior and, in particular, in the nucleus accumbens and the medial prefrontal cortex, as seen in RHA and RLA rats. Nonetheless, in view of the above recalled differences in baseline and novelty-evoked extracellular dopamine levels found in the nucleus accumbens of bNEHR and bNELR rats, it is tempting to speculate that changes in dopamine release also take place in the nucleus accumbens of male rats belonging to these two rat sub-lines during copulatory activity and that these changes are modified by the acquisition of sexual experience in these two rat sub-lines as well.

In comparison with RHA and RLA rats, it is also likely that modifications in dopamine activity similar to those found in the nucleus accumbens may also take place in other brain areas in which dopamine is involved in sexual behavior, e.g., the medial prefrontal cortex, medial preoptic area and the PVN of bNEHR and bNELR rats. In this regard, it is noteworthy that both extracellular dopamine and noradrenaline levels increase during copulatory behavior in the medial prefrontal cortex of both RHA and RLA rats in a rat line- (RHA rats show levels of both neurotransmitters higher than RLA rats) and sexual experience-dependent way (sexually experienced RHA rats show extracellular levels of both neurotransmitters higher than those of sexually experienced RLA rats) and with basal noradrenaline values in RHA rats about two-fold higher than those of RLA rats [255]. This finding resembles the higher values of basal noradrenaline found in the nucleus accumbens of bNEHR rats versus bNELR rats [277] and makes it likely that basal extracellular noradrenaline levels may also be higher in the nucleus accumbens of RHA versus RLA rats. Thus, further studies are required to verify whether alterations in dopamine function that may be related to the different copulatory activity of the bNEHR and bNELR rat sub-lines take place during copulation not only in the nucleus accumbens as ascertained in RHA and RLA rats, but also in other brain areas where dopamine influences sexual behavior, e.g., the medial preoptic area and the PVN of both RHA/RLA and bNEHR/bNELR/rat sub-lines.

### 6.7. LY and HY Rats, Erectile Function, Sexual Behavior and Dopamine

Low yawning (LY) and High Yawning (HY) rats are two Sprague-Dawley-derived rat sub-lines selectively bred for their different, low versus high, yawning frequency, with HY rats displaying a mean of 20 spontaneous yawns/hour, and LY rats 1–2 yawns/hour, respectively [224,225]. HY rats also display much more spontaneous penile erection episodes than LY rats, with a linear correlation between these two behavioral patterns. This correlation occurs also when the frequency of both penile erection and yawning is increased by the mixed dopamine D1/D2 receptor agonists (e.g., apomorphine and bromocriptine) [224] and the selective dopamine D2-like receptor agonist quinpirole [278], oxytocin [279], and when dopamine agonist-induced responses are abolished by metoclopramide, a mixed dopamine D1/D2-like receptor antagonist [224]. These findings resemble those found in RLA rats, which are more responsive to low doses of apomorphine and other selective D_2_ receptor agonists, being apomorphine able to induce a higher number of yawns [81,245] and penile erections in RLA than RHA rats [81]. Similar to those of RHA and RLA rats, bNEHR and bNELR rats also display the copulatory patterns of LY and HY rats (Table 14).

Accordingly, a very high number of HY rats do not copulate and reach ejaculation not only the first time (no sexually naïve HY rats reached ejaculation in the first two copulation tests), but also after several repeated exposures to a sexually receptive female rat (only 50% of HY rats reach ejaculation after eight tests) when compared to LY rats, 10% of which reached ejaculation in the first test and 80% after eight tests [280,281]. The failure of HY rats to copulate to ejaculation is believed to be secondary to deficits in recognizing sexually relevant odors [281]. In fact, these deficits may be responsible for a lower sexual motivation in these rats with respect to those with a normal perception of sexually relevant odors, as found in other non-copulator rats [281,282,283] or mice [284].

The lower sexual motivation and worse sexual performance together with the different response to dopamine agonists of HY versus LY rats (HY rats are more sensitive than LY rats to apomorphine and bromocriptine, see above) resemble the lower sexual motivation and worse sexual performance [247] and the higher response to the apomorphine facilitatory and haloperidol inhibitory effects on yawning, erectile function and sexual behavior of RLA versus RHA rats [82,245,247,253]. Since these differences between RHA and RLA rats depend on the different dopaminergic tone of the two Roman rat sub-lines (e.g., RHA rats display a stronger mesolimbic/mesocortical dopaminergic tone than RLA rats) (Section 6.5.1 and Section 6.5.2), it is tempting to speculate that a different central dopaminergic tone is also present in the LY and HY rat sub-lines, and that this plays a role in the copulatory patterns differences between LY and HY rats. Unfortunately, no data supporting this possibility is available so far; thus, further experiments on the LY and HY rat sub-lines are warranted to test this possibility.

### 6.8. Erectile Function and Sexual Behavior in Rats with a Genetically-Induced Increase in Dopaminergic Tone

The best known animals affected by genetic mutations that cause selective changes in the dopamine tone are those with silencing of the gene that synthesize the dopamine transporter (DAT) (which allows dopamine reuptake in nerve terminals, thus terminating the stimulation of dopamine receptors in the synaptic cleft) and which are considered an animal model of hyperdopaminergia [285,286,287,288,289,290], or the genes that synthesize dopamine D1/D2 receptors on which dopamine acts to induce its effects on cellular targets, and which are considered animal models of hypodopaminergia [291,292,293,294]. Although changes in dopamine tone should also occur when silencing one or more of the enzymes that control dopamine synthesis and content, such as tyrosine hydroxylase that synthesizes L-DOPA, the precursor of dopamine but also of noradrenaline (see Introduction, Section 1), or monoamine-oxydases (MAO A and B) that metabolize not only dopamine but also noradrenaline and serotonin (see [295] and references therein), silencing of these genes produce animals in which not only the dopamine but also noradrenaline and/or serotonin tones are profoundly modified, i.e., decreased dopamine and noradrenaline tone in tyrosine hydroxylase missing genotypes and increased catecholamine and serotonin tones in MAO missing genotypes.

The studies with mutant animals with DAT or D1/D2 receptor silencing recalled above may contribute to characterizing the role of dopamine in numerous central functions, from motility and movement control to motivation and reward, drug dependence and addiction and in mental pathologies, from psychosis to depression and others and even in copulatory behavior. Nonetheless, one work only was found in the literature which analyzes sexual behavior in recently developed DAT-KO rats (total gene silencing) compared to their Heterozygote (HET) (partial gene silencing) and Wild type (WT) (no gene silencing) counterparts [296]. These DAT-KO rats display numerous behavioral dysfunctions that have been related to the elevated extracellular dopamine levels and altered dopamine turnover caused by the DAT gene silencing present in these animals. Accordingly, they display marked spontaneous locomotor hyperactivity associated to cognitive impairments (i.e., working memory) and sensory-motor gating, which do not occur in the HET and WT animals. These rats show also impulsive/compulsive traits, stereotypies, anhedonia, asocial profile, alterations in coping with novelty and in motivation [297,298,299,300,301], with many of these traits resembling those that occur in RHA and bNEHR rats when compared to RLA and bNELR rats (see above). Apparently, these behavioral modifications are mainly related to the dysfunctional striatal dopamine turnover caused by the total or partial DAT gene silencing, which causes a marked increase in the dopaminergic tone across the central nervous system, and to alterations in frontostriatal BDNF, trkB and post-synaptic density protein 95 (PSD-95) levels. This led to consider these animals as a model for studying pathological hyperdopaminergic conditions, from the attention deficit/hyperactivity disorder (ADHD) to autism and psychosis spectrum disorders [290].

### 6.9. DAT-KO, DAT-HET and DAT-WT Rats, Sexual Behavior and Dopamine

In addition to the behavioral differences recalled above, DAT-KO rats also differ from their heterozygote (HET) and Wild Type (WT) counterparts when put for the first time in the presence of an inaccessible sexually receptive (ovariectomized and estradiol + progesterone-primed) female rat and when copulation is allowed in classical copulation tests [296]. In fact, in the first copulatory test, more than 80% of DAT-KO and HET rats initiate copulation with a sexually receptive female rat, displaying mounts and intromissions, but with 85% of DAT-KO rats reaching ejaculation versus 60% of HET rats, and 50–60% of WT rats initiating copulation and reaching ejaculation in the first test. DAT-KO and HET rats showing mounts, intromissions and reaching ejaculation raise to 100% in the second test, as for WT rats in the third test. The differences seen in the first and second test in the three rat sub-lines disappeared in the fourth and fifth test. Interestingly, the above differences occurred together with modifications in the copulatory patterns of DAT-KO, HET and WT rats along the five tests. In fact, statistical analyses of copulatory parameters of the first series of copulatory activity of the five copulatory tests, revealed significant differences in mount, intromission and ejaculation latencies, intromission and ejaculation frequency and post ejaculatory interval along the five tests among the three rat sub-lines (Table 15).

These differences confirm higher levels of sexual activity in DAT-KO and HET rats compared to WT rats and persist along the five tests. Accordingly, DAT-KO rats displayed shorter ejaculation latencies, higher ejaculation frequencies and shorter post ejaculatory intervals, when compared to WT and HET rats, being these differences more evident in the last two copulatory tests, when sexual experience has been acquired [296].

In comparison with what was discussed for RHA and RLA rats and bNEHR and bNELR rats, the differences in copulatory patterns among DAT-KO, HET and WT rats may be due to the different dopaminergic tone of the three rat sub-lines, with DAT-KO rats having much higher dopaminergic activity secondary to the DAT gene silencing when compared to HET rats and WT rats, the former with a partial and the latter with no DAT gene silencing. Accordingly, after five copulatory tests, the basal concentration of extracellular dopamine in the nucleus accumbens shell dialysate of DAT-KO rats is about five-fold and ten-fold higher than that of HET and WT rats, respectively. Perhaps more importantly, the concentration of extracellular dopamine in the nucleus accumbens shell dialysate increases differentially in DAT-KO, HET and WT rats when the animals are put in the presence of an inaccessible female rat and even more when copulation is allowed. As expected, extracellular dopamine increases in the nucleus accumbens shell dialysate in all the three rat sub-lines, for the whole copulation period and decreases after removal of the female rat. The increases in absolute dopamine values in the dialysate are much higher in DAT-KO rats than in HET and WT rats, in line with the higher basal extracellular dopamine levels of DAT-KO rats versus HET and WT rat sub-lines, respectively (Table 16 and Table 17) [296].

The above differences in extracellular dopamine in the nucleus accumbens dialysate found in DAT-KO, HET and WT rats took place in concomitance with modifications in sexual activity, as shown by the different number of non-contact penile erection episodes seen when the female rat was inaccessible and the changes in mount, intromission and ejaculation frequencies recorded during copulation in the three rat sub-lines. Accordingly, (i) HET rats displayed more non-contact penile erections than DAT-KO and WT rats as well; (ii) HET and, to a lesser extent, DAT-KO rats displayed more mounts than WT rats; and (iii) DAT-KO rats displayed more ejaculations (higher ejaculation frequency) than WT and, to a lesser extent, HET rats. In addition, in line with the findings of the last two tests done before microdialysis: (i) DAT-KO and HET rats show lower mount, intromission and ejaculation latencies than WT rats; (ii) HET rats show higher and DAT-KO rats lower mount frequency than WT rats; and (iii) DAT-KO rats show lower intromission frequency and higher ejaculation frequency than WT and HET rats, but to a lesser extent (Table 15). Together, these results indicate a higher rate of approaching behavior to female rats and thus higher sexual motivation and better sexual performance of DAT-KO and HET rats versus WT rats during the whole experiment, in line with the higher dopaminergic tone of DAT-KO and HET versus WT rat sub-lines [296].

## 7. Dopaminergic Systems, Sexual Motivation, Reward and Performance

The studies reviewed above show that central dopaminergic systems play a main role in both the anticipatory/appetitive (motivation, arousal and reward) and/or consummatory (erectile function and sexual performance) phases of male sexual behavior, although with different relevance in these two phases among them. In fact, although it is commonly accepted that mesolimbic/mesocortical dopaminergic neurons are those playing the main role in the anticipatory components of male sexual behavior [69,141,142,198,250,257], several lines of experimental evidence also show that incertohypothalamic dopaminergic neurons innervating the medial preoptic area and the PVN, which are believed to play a main role in erectile function and sexual performance, are also involved in sexual motivation (see [140,161,260]). The main role of mesolimbic/mesocortical dopaminergic neurons in sexual motivation, arousal and reward comes from studies which demonstrate an important role of these neurons in motivational, emotional and rewarding processes [141,142,143,172,250,256,257,258,259,260]. In the 1990s, numerous studies were dedicated to the involvement of mesolimbic dopaminergic neurons in sexual motivation as opposed to the facilitatory role of nigrostriatal dopaminergic neurons in sexual performance. The different, but complementary role of these two dopaminergic systems was clarified by using the bi-level chambers (designed by Mendelson and Gorzalka, 1987 [170]), which allow for discriminating the appetitive from the consummatory phase of copulatory behavior and to quantify their various components. In the bi-level chamber, the male rat chases the female from one level to another after each intromission, and the number of level changes in a fixed time before the introduction of the female represents a measure of the appetitive phase of sexual behavior. By means of this procedure, D1 or D2 receptor antagonists administered systemically or injected into the nucleus accumbens or the medial preoptic area were found able to decrease male rat levels changing given at doses much lower than those that decrease copulatory activity, as indicated by the increases in mount, intromission and ejaculation latency and post ejaculatory interval and the decrease in ejaculation frequency and in sensory-motor performance [172,198]. These findings support the hypothesis that (i) dopamine receptor antagonists impair mainly the appetitive/preparatory phase of copulatory behavior rather than its consummatory aspects once sexual activity was initiated, and (ii) the impairment of copulatory activity reported after high doses of these compounds may be secondary to the impairment of motor performance by the blockade of striatal dopamine receptors, rather than sexual motivation [193,198,302]. These conclusions were also supported by instrumental methods, in which the male rat learns to press a lever in order to gain access to a receptive female after a conditioning stimulus and copulate with her (see [303]) or to choose a place where it previously copulated or a receptive female was placed (place preference) [147]. Accordingly, (i) the mixed D1/D2 receptor antagonist α-flupenthixol given systemically decreased the ability of the animals to press the lever (e.g., their motivation towards sex), at doses much lower than those that impair copulation [303] and (ii) D-amphetamine infused into the nucleus accumbens but not in the striatum significantly decreased mount and intromission latency of intact, sexually potent male rats and restored lever pressing to reach the receptive female rat in male rats with excitotoxic lesions of the basolateral amygdala [303,304].

The main involvement of mesolimbic/mesocortical dopaminergic neurons in the anticipatory/appetitive phases of copulatory behavior and in sexual arousal/motivation as well was confirmed by biochemical studies showing that dopamine neurotransmission (measured by L-DOPA accumulation after central amino acid decarboxylase inhibition) is activated more in the nucleus accumbens compared to the striatum during the anticipatory phase of copulatory behavior of sexually naive male rats with a sexually receptive, but not non-receptive female rat or in sexually experienced male rats with a receptive female rat [305,306,307]. Increased dopamine activity during copulatory behavior was also suggested by increased dopamine and DOPAC levels found in the preoptic region and lumbosacral spinal cord of male rats [256,260,262,263,308]. Although supporting the hypothesis that mesolimbic dopaminergic neurons play a main role in the anticipatory phase of sexual behavior, the above studies did not reveal how dopamine activity is modified in the different areas of the brain during the two main phases of sexual behavior. This is important for ascertaining if the changes in neuronal activity were taking place during the anticipatory or the consummatory phase of copulatory behavior and/or in concomitance with a given aspect of the copulatory activity. This was obtained by the using methods such as differential pulse voltammetry, chronoamperometric and mainly microdialysis techniques, which allow one to follow changes in the activity of a given neurotransmitter (i.e., release of the neurotransmitter itself or of its metabolites) in selected brain areas of rats previously implanted with electrodes or microdialysis probes during the experimental procedure (e.g., during copulatory activity). As extensively reviewed in the above sections, microdialysis experiments have definitively confirmed that (i) dopamine and its metabolites DOPAC and homovanillic acid (HVA) levels increase in the nucleus accumbens dialysate from sexually potent male rats put in a test chamber containing only odors of a previous copulation and divided into two parts with a removable wire-mesh screen [142], (ii) the dopamine increase was further augmented first by the introduction of a receptive female behind the wire-mesh screen and second when the wire-mesh screen was removed and copulation allowed, (iii). dopamine in the striatal dialysate increased much more slowly becoming significant only during copulation [142] and finally (iv) the dopamine increase was not related to novelty or locomotion, since in these experimental conditions dopamine levels increased only in the striatum [142]. Microdialysis studies also revealed an increased dopamine activity in the anticipatory phases of copulatory behavior and a further increase during sexual interaction not only in the nucleus accumbens and, to a lesser extent, in the striatum as reported above, but also in the medial preoptic area [260,261,262] and in the PVN [140] of sexually potent male rats. In these studies, dopamine and metabolites were also increased in the presence of an inaccessible receptive female rat and much more during copulation and declined after ejaculation [140,260,261,262]. Interestingly, dopamine D2-like receptor agonists injected into the PVN at doses that induce penile erection, also increase extracellular dopamine levels in the nucleus accumbens dialysate, being that this increase was abolished by the prior blockade of oxytocinergic receptors in the ventral tegmental area [80,155]. The latter results reveal an important interaction between the activity of incertohypothalamic and mesolimbic dopaminergic neurons that plays a role in the facilitatory effect of dopamine in erectile function and sexual behavior, and are in line with the existence of a neural circuit that participates in the control of both the anticipatory and consummatory phases of sexual behavior [80,101,102,155,161] (Section 7 and Section 8).

Altogether, the studies reviewed above confirm and extend the results of earlier studies that support the hypothesis that the mesolimbic dopaminergic system plays a main role in the anticipatory/motivational phase, whereas the nigrostriatal dopaminergic system supplies the sensory-motor coordination necessary for the consummation of the behavior. In addition, these studies also show that the incertohypothalamic dopaminergic neurons are involved in sexual motivation, possibly by interacting with mesolimbic dopaminergic neurons. Finally, the results of these studies provide evidence that copulatory behavior is also rewarding and that its rewarding effects are mediated by increased dopamine release in the nucleus accumbens, as with that of other rewarding stimuli, i.e., feeding, drinking and self-stimulation [141,142,143,172,250,303,308,309].

## 8. Final Remarks

The studies reviewed in this work confirm and extend the results of earlier studies suggesting that dopamine exerts a facilitatory role in erectile function and sexual behavior in male rats. Since in male rats mixed dopamine and D2-like receptor agonists induce penile erection (the key consummatory component of male sexual activity), and among the main effects of these compounds on copulation are either a decrease in mount and intromission latency and an increase in ejaculation frequency, dopamine was assumed to improve both sexual motivation and sexual performance in male rats. Accordingly, among the copulatory parameters measured (mount, intromission and ejaculation latencies, number of intromissions and of ejaculations, post ejaculatory interval), changes in mount latency only are considered to reflect modifications in sexual motivation (e.g., an increase or a decrease in this parameter indicates an increase or a decrease in sexual motivation, respectively), whereas the other parameters provide only insights on the consummatory phase of the behavior, or on the rate at which copulation takes place. As recalled above (Section 5, Section 5.1 and Section 5.2), the facilitatory involvement of dopamine in sexual motivation was confirmed by using either the bi-level chambers of Mendelson and Gorzalka [170], which allow discriminating the anticipatory from the consummatory phase of sexual behavior and quantifying their different components [114,139], or with instrumental methods, in which the male rat learns to press a lever to gain access to a receptive female after a conditioning stimulus to copulate with her (see [300,301,307]), or to choose a place where it had previously copulated or where a receptive female was placed [147,172,198,303,304,309,310]. In fact, these studies provide clear evidence that blockade of central dopaminergic receptors in sexually relevant brain areas, e.g., the nucleus accumbens, PVN and medial preoptic area, which do not interfere with motor and posture control, impairs sexual behavior by reducing sexual motivation and arousal rather than sexual performance.

**Figure 3:**
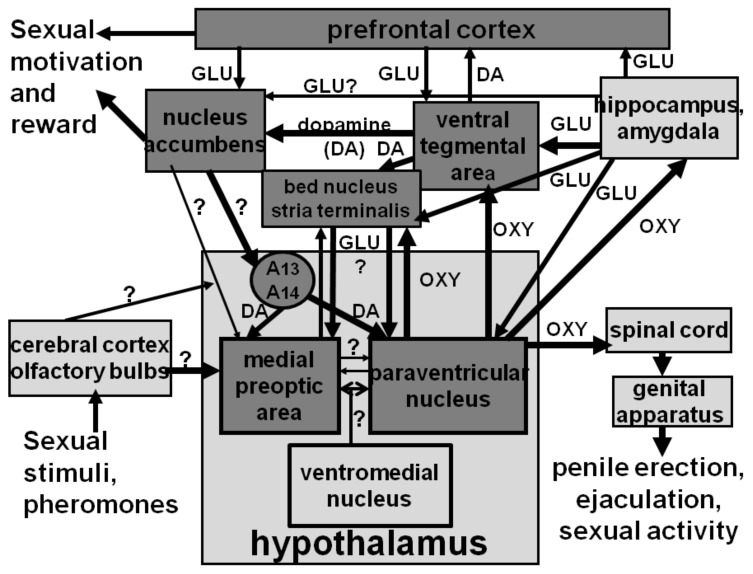
Dopamine participates in a complex central neural circuit that controls and influences sexual motivation and reward and copulatory performance at the central level together with other neurotransmitters and/or neuropeptides. This neural circuit interconnects the hypothalamus and its nuclei (paraventricular and ventromedial nuclei) with areas of the limbic system (ventral tegmental area, hippocampus, amygdala, bed nucleus of the stria terminalis), with the ventral medulla and the spinal cord. Among involved dopaminergic pathways are (i) incertohypothalamic dopaminergic neurons, which activate PVN oxytocinergic neurons that send their projections to the ventral medulla and spinal cord and to the ventral tegmental area, hippocampus, amygdala and bed nucleus of the stria terminalis; (ii) mesolimbic/and mesocortical dopaminergic neurons that originate in the ventral tegmental area that send projections to the nucleus accumbens, medial prefrontal cortex, bed nucleus of the stria terminalis and possibly to the hippocampus and amygdala. Together with glutamatergic neurons that interconnect the hippocampus and the amygdala with the ventral tegmental area, prefrontal cortex, bed nucleus of the stria terminalis, medial preoptic area and paraventricular nucleus, when this circuit is activated for instance by physiological stimuli (i.e., pheromones released by a sexually receptive female) or by drugs or peptides administered in one of the areas of the circuit (i.e., oxytocin injected into the ventral tegmental area, the hippocampal ventral subiculum or the amygdala), dopamine becomes active at different sites, i.e., in the paraventricular nucleus and the medial preoptic area, which are involved more in copulatory performance (penile erection and copulation), and in the nucleus accumbens in which dopamine plays a main role in sexual motivation, arousal and reward (heavy gray boxes indicate the brain areas of the circuit in which dopamine is involved/released). Thus, modifications in dopamine activity in these brain areas participate in the regulation of sexual motivation/arousal/reward and copulatory performance (erectile function/copulation) (for references see [82,100,102]).

The role of dopamine in both sexual arousal and motivation and in sexual performance was definitively confirmed when extracellular dopamine and its metabolites were found increased in the dialysate from the nucleus accumbens [141,142,143] as well as from the medial preoptic area and the PVN of male rats [80,140,260,262], not only when exposed to an inaccessible receptive female rat, e.g., when male rats display non-contact erections that are considered as an index of sexual arousal (see [90,140,311] and references therein), but also, and even more, during copulation [140,162,254,260,262]. Together, these studies confirm that dopamine improves erectile function and sexual performance by acting in different brain areas, i.e., the nucleus accumbens and the medial prefrontal cortex, in which dopamine is released by mesolimbic/mesocortical dopaminergic neurons whose cell bodies are localized in the ventral tegmental area, and the bed nucleus of the stria terminalis, which also receives dopaminergic projections from the ventral tegmental area ([162,163] and references therein). These dopaminergic neurons, which play a major role in motivational and rewarding processes activated by natural stimuli, such as food, water and sex itself (see [303,304,310]), are extensively connected through many neuronal systems containing either neurotransmitters (i.e., glutamic acid, nitric oxide) and/or neuropeptides (i.e., oxytocin, opioid peptides) (see [100,101,102,151,161]) with other brain areas involved in the control of erectile function and copulatory behavior. These include the medial preoptic area, the hypothalamus and its nuclei (i.e., the PVN), which send neural projections controlling erectile function not only to the spinal cord but also to extra-hypothalamic brain areas such as the ventral tegmental area, hippocampus and amygdala. Interestingly, both the medial preoptic area and the PVN contain dopaminergic nerve endings, being innervated by incertohypothalamic neurons [120,121] and activation of dopaminergic receptors in these areas facilitates penile erection and copulatory behavior. Finally, the PVN sends oxytocinergic projections not only to the spinal cord but also to the ventral tegmental area, hippocampus and amygdala (see [101] and references therein). In the ventral tegmental area, oxytocinergic nerve terminals make contact with the cell bodies of mesolimbic/mesocortical dopaminergic neurons [155,156], which also receive direct or indirect (through the medial prefrontal cortex) excitatory glutamatergic projections which originate in the hippocampus and the amygdala ([157,158] and references therein), and which are also activated by oxytocinergic neurons that originate in the PVN [159,160]. In line with the above findings, the stimulation of dopamine D2-like receptors in the PVN by dopamine receptors agonists (i.e., apomorphine) induces penile erection and increases extracellular dopamine in the nucleus accumbens, and both these responses are markedly reduced by the oxytocinergic receptor antagonist d(CH_2_)_5_Tyr(Me)^2^-Orn^8^-vasotocin given intracerebroventricularly [80] or into the ventral tegmental area [157]. Together, the studies reviewed above suggest that a neural circuit connects the PVN and possibly the medial preoptic area, with the ventral tegmental area, the hippocampus, the amygdala and the nucleus accumbens and from these areas through unknown pathways back again to the PVN in order to regulate the activity of oxytocinergic neurons projecting either to the spinal cord (mediating penile erection) or to the ventral tegmental area and/or the hippocampus, the amygdala and the bed nucleus of the stria terminalis (to regulate the activity of mesolimbic/mesocortical dopaminergic neurons) (see Figure 3). It is feasible to assume that this neural circuit, in which numerous neurotransmitters (dopamine, glutamic acid, nitric oxide) and neuropeptides (oxytocin, opioid peptides) participate and interact with each other, is involved in the integration of neural activities controlling consummatory (erectile–ejaculatory) and anticipatory (motivational and rewarding) aspects of copulatory behavior in physiological contexts. Accordingly, the levels of extracellular dopamine and its metabolite DOPAC increase in the nucleus accumbens (see above) and in the PVN as well of sexually active male rats in the presence of an inaccessible receptive female rat, when noncontact erections take place, and even more during copulation (e.g., when in copula penile erections take place) [140]. Thus, this neural circuit contributes at the same time to the consummatory aspects of sexual behavior and may also influence mesolimbic/mesocortical dopaminergic neurons supplying a neural substrate to explain the well-known rewarding properties of sexual behavior (see [250,303,311,312]). In fact, as recalled above, mesolimbic/mesocortical dopaminergic neurons have a key role in the motivational and rewarding properties of the natural reinforcing stimuli, such as food, water and sexual behavior (see [303,313,314]). In particular, dopamine released from these neurons is thought to be involved in the transposition of the motivational aspects of natural stimuli into goal directed behaviors, as it may be the seeking of a sexual partner and of sexual intercourse to reach reward and satisfaction for sexual activity, [315].

More recent studies on this matter show that the extracellular dopamine increase in the nucleus accumbens and medial prefrontal cortex is correlated to the dopaminergic tone of the male rats, which determines their pattern of copulatory performance. Accordingly, rat sub-lines with a grater dopaminergic tone than their control counterparts obtained by selective breeding protocols over multiple generations for showing opposite specific behavioral tracts (i.e., RHA versus RLA rats, bNEHR versus bNELR rats), a specific spontaneous behavioral response (LY versus HY rats) or by genetic mutations (DAT-KO versus HET and WT rats) show improved erectile function and copulatory behavior and specific patterns of copulation that occur in parallel with changes in dopamine activity in the nucleus accumbens and in the medial prefrontal cortex when compared to their lower dopaminergic tone counterparts [255,296,316].

The fact that rat sub-lines psychogenetically selected for showing specific (often opposite and contrasting) traits or even to show a spontaneous behavior or not, over multiple generations, show similar differences in sexual behavior and that a different dopaminergic tone present in these rat sub-lines, which resembles that of DAT-KO rats versus HET and WT rats, plays a main role in these differences deserves some comment.

First, it is feasible that the behavioral differences between the above rat sub-lines are related to genetic and environmental factors, which concur with modifications in sexual behavior, related to alterations in the function of brain neurotransmitters (e.g., dopamine, although other neurotransmitters may be involved, such as noradrenaline and also serotonin).

This raises the possibility that, depending on the behavioral criteria on which the psychogenetic selection is based, phenotypes may be generated that are strongly related to each other not only in behavioral traits but also in sharing central and/or peripheral neural and neuroendocrine substrates of their different behavioral patterns. This makes psychogenetically selected animal sub-lines important animal models to study associations between neurotransmitters function and behavior, including sexual behavior in physiological conditions or in the presence of brain lesions, mental illness or after the administration of psychoactive drugs [222,316]. In this regard, it is really noteworthy that among neurotransmitters playing a major role in sexual behavior and whose function (tone) is modified in the rat sub-lines reviewed here, dopamine is one of the most involved. In fact, this is in agreement not only with the findings of the reviewed studies that show dopamine as a key neurotransmitter in copulatory behavior, but also with the studies that show that rats in which dopamine synthesis and dopamine receptors are eliminated by knocking out tyrosine hydroxylase and dopamine receptor (of the D1 and the D2 families) genes show a hypoactive phenotype (see [317]), whereas the DAT gene knockout rats display a hyperactive phenotype [290] with increased sexual motivation and better sexual performance than their WT counterparts, as discussed above.

Second, it is also likely that associations between dopamine function and alterations in copulatory behavior observed in these rat sub-lines, also take place in humans. Although it is difficult to translate rodents’ sexual behavior parameters to human sexual behavior, this is highly likely and would be also not surprising (see [318]). Indeed, (i) a relationship between hypersexual behavior (e.g., excessive sexual behavior associated with decreased ability of a person in controlling his/her sexual behavior) and personality traits has been long discussed in research and clinical practice (see [319] and references therein), and (ii) relations between trait impulsivity (i.e., sensation seeking), behavioral impulsivity (i.e., behavioral risk-taking) and high levels of sexual arousal and risky sexual behavior [that lead to negative long term consequences, e.g., unplanned pregnancy, human immunodeficiency virus (HIV) transmission and other sexually transmitted diseases] have been often reported among young men and women ([320,321] and enclosed references).

Third, although many neurotransmitters and/or neuropeptides (dopamine, serotonin, oxytocin and others) may play a role in the determination of trait and behavioral impulsivity, excessive levels of sexual arousal and hypersexuality, hypersexuality and paraphilias (which are characterized by recurrent, intense, sexually arousing fantasies, urges, or behaviors, often involving non-human objects, suffering or humiliation of oneself or one’s partner, or children or other non-consenting persons) can be observed in Parkinson’s disease patients treated with dopaminomimetic drugs (from L-DOPA to direct dopamine receptor agonists) with the severity of these symptoms decreasing by reducing the dose of or by discontinuing the dopaminomimetic drugs used (see [322] and references therein). Thus, in spite of the limitations raised by the difficulties in translating rodents’ sexual behavior to human sexual behavior, it appears feasible to translate the finding that rats selectively bred to show opposite modifications in some behavioral traits also display parallel modifications in copulatory behavior, although with due attention to the manifestation of divergent behavioral traits and associated alterations in sexual behavior in humans. In fact, it is impossible not to recall that rats are a predictive model of human sexual function and that many of the now available treatments for sexual dysfunctions in men and women (including phosphodiesterase type V inhibitors and dopaminergic agonists) have been discovered in rats (see [83,151,319] and references therein).

In conclusion, the studies reviewed in this work confirm also that the facilitatory role of dopamine in erectile function and copulatory behavior is mediated mainly by dopamine receptors of the D2 family and show in particular that dopamine D_2_ receptors are those that control erectile function and the facilitation of male sexual behavior induced by mixed D1/D2 dopamine receptor agonists given systemically, although a role of dopamine D1 receptors, usually opposite to that of D2 receptors, has also been found to occur in some brain areas (e.g., the medial preoptic area) [48]. However, D_4_ receptors are also involved, since (i) selective D_4_ receptor agonists induce penile erection when given systemically or into the PVN [79,155], and (ii) when given systemically D_4_ receptor agonists also facilitate male rat copulatory behavior, an effect that is abolished by the prior treatment with selective D_4_ receptor antagonists, which exert only minor effects on the improvement of copulatory behavior induced by mixed D1/D2 dopamine receptor agonists [70,151]. The facilitatory effect of selective D_4_ receptor agonists on erectile function and the copulatory behavior found in male rats may be the basis of a new pharmacological strategy for the therapy of erectile dysfunction in men. Accordingly, a few selective D_4_ dopamine receptor agonists (i.e., ABT 724), given at doses found able to induce penile erection and to facilitate sexual behavior in male rats [58,70], have been found ineffective in inducing vomiting in the ferret, a common test usually used to evaluate nausea and emetic properties of drugs [153]. If both these finding were also confirmed in humans, D_4_ receptor agonists devoid of emetic properties are certainly worthy to be tested for their clinical use in men affected by erectile dysfunction, as already done for the mixed D1/D2 receptor agonist apomorphine. As recalled above, a formulation for the sublingual administration of this compound was made commercially available in the 1990s for the therapy of erectile dysfunction, but the success of this formulation was scarce (in particular if compared to that of orally active phosphodiesterase type V inhibitors) due to the emetic properties of this compound, which are incompatible with sexual intercourse [83,151]. Thus, the availability of safe and selective dopamine D_4_ receptor agonists that facilitate erectile function but are devoid of emetic properties would result in a new strategy for the treatment of erectile dysfunction in men.

## Figures and Tables

**Table 1:** Dopaminergic systems in the central nervous system.

Dopaminergic System	Localization of Neuronal Cell Bodies	Localization of Nerve Endings	Functions
nigrostriatal	substantia nigra (A9 group)	striatum	motor and posture control
mesolimbic	ventral tegmental area (A10 group)	nucleus accumbens	motivation, arousal, reward
mesocortical	ventral tegmental area	medial prefrontal cortex	motivation, attention, decision making
incertohypthalamic	hypothalamus (dopaminergic A13–A14 groups)	hypothalamic nuclei (PVN) and medial preoptic area	different functions (feeding, erectile function, sexual behavior)
tuberoinfundibular	hypothalamus-arcuate nucleus	median eminence	inhibition of prolactin release by pituitary lactotropes

Details are in References [9,12,22,23,24,25,26,27,28,29,30,31].

**Table 2:** Classification of dopamine receptors and their role in penile erection and copulatory behavior.

Dopamine Receptor Families	Transduction Mechanisms	Receptor Subtypes	Role in Penile Erection	Role in Copulatory Behavior
D1	increased activity of adenylate cyclase-cAMP signaling pathway, increased PIP_2_ turnover, increased Ca^2+^ mobilization	D_1_	none (but see [48])	none (but see [49,50])
		D_5_	not available	not available
D2	decreased activity of adenylate cyclase-cAMP signaling pathway, increased K^+^ channels activation, increased voltage-gated Ca^2+^ channels activation	D_2s_–D_2l_	facilitatory	facilitatory
		D_3_	none (but see [51])	facilitatory *
		D_4_	facilitatory (but see [51,52])	facilitatory

Details are in References [12,13,14,15,16,27,28,53,54]. * Facilitation of ejaculation processes [53,54].

**Table 3:** Binding affinity characterization of D2-like receptor agonists and antagonists by in vitro studies on the D_2_, D_3_ and D_4_ receptors.

	Binding Affinities at			References
	D_2_ Receptors	D_3_ Receptors	D_4_ Receptors	
	*K*i Values (nM)			
**D2-like receptor agonists**				
apomorphine	32	26	2.6	[55]
quinpirole	1.8	0.96	3.0	[56]
pramipexole	3.9	0.5	5.1	[56]
PD 128,907	931	9.7	2430	[51]
PNU-95,666E	144	493	>10,000	[51]
PD 168,077	3740	2810	8.7	[57]
ABT-724	>10,000	NR	63.6	[58]
PIP-3EA	990	3900	2.8	[59]
FAUC 3019	33	82	0.4	[60]
FAUC 389	210	390	1.5	[61]
A-412997	2848	2095	7.9	[62]
CP 226269	1760	NR	6.0	[63]
**D2-like receptor antagonists**				
L-741,626	2.4	100	220	[64]
SB277011A	1000	10	not available	[65]
FAUC 365	3600	0.5	340	[66]
L-745,870	960	2300	0.43	[64]
haloperidol	6.3	6.1	10	[67]
raclopride	1.0	1.3	5070	[68]

**Table 4:** Effect of old and new dopamine receptor agonists on penile erection and copulatory behavior.

Dopamine Receptor Agonist	Dopamine Receptor Involved	Effect on Penile Erection	Effect on Copulatory Behavior	References
Apomorphine	D_1_/D_2_/D_3_/D_4_/D_5_	facilitatory	facilitatory	[46,47,55,69,70]
SKF 38393	D1	none	not available	[46,71,72,73]
Quinpirole	D_2_/D_3_/D_4_	facilitatory	facilitatory	[51,69]
Pramipexole	D_3_/D_2_	facilitatory	facilitatory	[51,74,75,76,77]
PD 128,907	D_3_/D_2_	facilitatory	not available	[51,52]
PNU-95666E	D_2_	facilitatory	not available	[51]
PD 168,077	D_4_	facilitatory	facilitatory	[55,74,75,78,79,80,81]
ABT-724	D_4_	facilitatory	facilitatory	[58,70]
PIP3EA	D_4_	facilitatory	not available	[59,79]
FAUC 3019	D_4_	facilitatory	not available	[60]
FAUC 389	D_4_	facilitatory	not available	[61]
A-412997	D_4_	facilitatory	not available	[62]
CP 226269	D_4_	facilitatory	not available	[55]

**Table 5:** D_2_ receptors mediate the proerectile effect of the mixed D1/D2 dopamine receptor agonist apomorphine and of the D_3_/D_2_ dopamine receptor agonist pramipexole but not of the D_4_ dopamine receptor agonist PD 168,077.

Pre-Treatment	Treatment			
	Saline	Apomorphine	Pramipexole	PD 168,077
	Effect on Penile Erection			
Saline	==	↑	↑	↑
L741,626	==	↓	↓	↑
SB277011A	==	↑	↑	↑
FAUC365	==	↑	↑	↑
L745,870	==	↑	↑	↓
Raclopride	==	↓	↓	↑

Apomorphine is a mixed D1/D2 agonist, pramipexole a D_3_/D_2_ agonist, PD 168,077 a D_4_ agonist; L741,626 a D_2_ antagonist, SB277011A and FAUC365 two D_3_ antagonists, L745,870 a D_4_ antagonist, raclopride a D_3_/D_2_ antagonist. The dopamine agonists were all injected subcutaneously, and the antagonists were given subcutaneously except L745,870 and raclopride that were given intraperitoneally 15 min before the agonists, (== = no effect; ↑ = increase; ↓ = decrease). Experimental details and data are in [58,59,60,61,74,75,78,79,80].

**Table 6:** D_2_ and D_4_ receptor agonists induce penile erection by activating nitric oxide synthase containing paraventricular oxytocinergic neurons that project to extrahypothalamic brain areas and the spinal cord.

Pre-Treatment	Treatment					
	Apomorphine		PD 168,077		ABT 724	
	Effect on					
	Penile Erection	NO Production	Penile Erection	NO Production	Penile Erection	NO Production
Saline	↑	↑	↑	↑	↑	↑
L741,626	↓	↓	↑	↑	↑	↑
L745,870	↑	↑	↓	↓	↓	↓
Oxy-Ant	↑	↑	↑	↑	↑	↑
Oxy-Ant I.C.V.	↓	↑	↓	↑	↓	↑
L-NAME	↓	↓	↓	↓	↓	↓
ω-Conotoxin	↓	↓	↓	↓	↓	↓

Apomorphine is a D1/D2 agonist, PD 168,077 and ABT 724 are two D_4_ agonists, L741,626 a D_2_ antagonist, L745,870 a D_4_ antagonist, raclopride a D_3_/D_2_ antagonist, Oxy-Ant is the oxytocin receptor antagonist d(CH_2_)_5_-Tyr (Me)^2^-Orn^8^-vasotocin, L-NAME is the nitric oxide (NO) synthase inhibitor nitro-L-arginine-methylester, ω-conotoxin is a N-type Ca^2+^ channel antagonist. All treatments were done in the PVN except when otherwise indicated (e.g., I.C.V.), with the antagonists given 15 min before the agonists. (== = no effect; ↑ = increase; ↓ = decrease). Experimental details and data are in [75,78,79,80].

**Table 7:** Dopamine released in the nucleus accumbens plays a role in penile erection induced either by oxytocin given to the ventral tegmental area, the ventral hippocampus and the amygdala or by D_2_ and D_4_ dopamine receptor agonists given to the PVN.

Pretreatment	Treatment	Penile Erection	Dopamine Release in the NAc
Vehicle	Oxytocin into the VTA, VS, or Am	↑	↑
Oxytocin antagonist into the VTA	Oxytocin into the VTA, VS, or Am	↓	↓
Dizolcipine into the VTA	Oxytocin into the VS and Am	↓	↓
Haloperidol into the NAc	Oxytocin into the VTA	↓	↑
Vehicle	Apomorphine into the PVN	↑	↑
Vehicle	PD 168,077 into the PVN	↑	↑
Oxytocin antagonist into the VTA	Apomorphine into the PVN	↓	↓
Oxytocin antagonist into the VTA	PD 168,077 into the PVN	↓	↓

Apomorphine is D1/D2 agonist, PD 168,077 a D_4_ agonist, haloperidol a D2-like receptor antagonist, dizolcipine [(+)MK801] is a NMDA receptor antagonist, oxytocin antagonist is d(CH_2_)_5_Tyr(Me)^2^-Orn^8^-vasotocin. Pretreatments and treatments were done in the areas indicated with the pretreatments done 15 min before the treatments. NAc = nucleus accumbens, VTA = ventral tegmental area, VS = ventral subiculum of the hippocampus, Am = amygdala, PVN = paraventricular nucleus. == = no effect; ↑ = increase; ↓ = decrease). Experimental details and data are in [80,155,156,157,158,159,160].

**Table 8:** Changes in copulatory behavior of sexually potent male rats induced by the D_4_ receptor agonists PD 168,077 and ABT-724: comparison with apomorphine.

Treatments	ML	IL	EL	MF	IF	EF	PEI
PD 168,077	==	==	==	↓	==	↑	↓
ABT-724	==	==	==	↓	==	↑	↓
Apomorphine	== *	== *	↓	↓	↓	↑	==

Dopamine receptor agonists were given to male rats ten minutes before the introduction of a sexually receptive female rat into the mating cage and copulatory parameters measured for 60 min. Changes refer to rats treated subcutaneously with saline (controls). ML, IL and EL = mount, intromission and ejaculation latencies (e.g., the time interval between the introduction of the female in the mating arena and the first mount/intromission/ejaculation, respectively); MF and IF = mount and intromission frequencies (e.g., number of mounts/intromissions in the first series of copulatory activity), EF =ejaculation frequency, the number of ejaculation in the entire copulatory test; PEI = post ejaculatory interval, the time interval between the first ejaculation and the first mount of the second series of copulatory activity. == = no effect; ↑ = increase; ↓ = decrease). Experimental details and data are in [70]. * Note that in [70], no significant effect of apomorphine on ML and IL was found against the decrease in both parameters reported by other studies reviewed above [35,36,195]. This discrepancy might be due to differences in the number of rats used in these studies.

**Table 9:** Changes in copulatory behavior of sexually potent male rats induced by the D_4_ receptor antagonist L-745,870: comparison with haloperidol.

Treatments	ML	IL	EL	MF	IF	EF	PEI
L-745,870	==	↑	↑	↑	==	↓	↑
Haloperidol	↑	↑	↑	==	↓	↓	↑

L-745,870 or haloperidol was given to male rats fifteen minutes before the introduction of a sexually receptive female rat into the mating cage and copulatory parameters measured for 60 min. Changes are referred to rats treated subcutaneously with saline (controls). ML, IL and EL = mount, intromission and ejaculation latencies; MF and IF = mount and intromission frequencies; EF = ejaculation frequency; PEI = post ejaculatory interval (for definitions see legend of Table 8). == = no effect; ↑ = increase; ↓ = decrease). Experimental details and data are in [70].

**Table 10:** Effect of the dopamine D_4_ receptor antagonist L-745,870 on dopamine agonist-induced facilitation of copulatory behavior of sexually potent male rats.

Pretreatment	Treatment	ML	IL	EL	MF	IF	EF	PEI
Vehicle	PD 168,077	==	==	==	↓	==	↑	↓
L-745,870	Vehicle	==	↑	↑	↑	==	↓	↑
L-745,870	PD 168,077	==	==	↑	==	==	==	==
Vehicle	ABT 724	==	==	==	↓	==	↑	↓
L-745,870	ABT 724	==	==	↑	==	==	==	==
Vehicle	Apomorphine	== *	== *	↓	↓	↓	↑	==
L-745,870	Apomorphine	==	↑	↓	↓	↓	↑	==

Pretreatments were given to male rats 15–20 min before treatments with dopamine agonists. Ten min after the pretreatment, a sexually receptive female rat was put into the mating cage and copulatory parameters measured for 60 min. Changes are referred to rats treated with saline (controls). ML, IL and EL = mount, intromission and ejaculation latencies; MF and IF = mount and intromission frequencies, EF = ejaculation frequency; PEI = post ejaculatory interval (for definitions see legend of Table 8). == = no effect; ↑ = increase; ↓ = decrease). Experimental details and data are in [70]. * Note that in [70], no effect of apomorphine on ML and IL was found against the decrease in both parameters reported by other studies reviewed above [35,36,195]. This discrepancy might be due to differences in the number of rats used in these studies.

**Table 11:** Mean values of copulatory parameters measured in the first series of copulatory activity of male RHA/RLA, bNEHR/bNELR rat sub-lines at the first (sexually naïve condition) and at the fifth copulatory test (sexually experienced condition) with a sexually receptive female rat.

	Psychogenetically Selected Rat Sub-Lines							
Copulatory Parameters	RHA		RLA		NEHR		NELR	
	Naive	Experienced	Naive	Experienced	Naive	Experienced	Naive	Experienced
NCPE	2.0	2.4 *	1.1 #	1.3 #	na	na	na	na
ML	290	25 *	600 #	400 * #	180	20 *	410 #	200 * #
IL	320	30 *	620 #	350 * #	190	20 *	520 #	200 *
EL	1200	500 *	1280	980 * #	830	570 *	750	580 *
MF	12	6 *	7 #	6	18	11 *	10 #	9
IF	20	20	10	10	15	15	10	9
PEI	360	300 *	480	450 #	280	330	330	320
EF	1.3	3.2 *	1	1.7 * #	na	na	na	na
III	62	30 *	120 #	76 * #	58	40 *	85 #	60 * #
CE	0.60	0.78 *	0.57	0.68 * #	0.60	0.65	0.55	0.57 #

NCPE = non-contact penile erections; ML, IL, EL = mount/intromission/ejaculation latency (seconds); MF, IF, EF = mount/intromission/ejaculation frequency; PEI = post ejaculatory interval (seconds) (for definitions see legend of Table 8); III = inter intromission interval (seconds), the ratio between the ejaculation latency of the first series and the number of intromissions in the series; CE = copulatory efficiency, the number of intromissions of the first series divided by the sum of the number of mounts and of intromission in the series. Experimental details and data for RHA/RLA rats are in [247] and for NEHR/NELR rats in [248]. na = not available. * = significantly different, experienced vs. naive, same rat sub-line, for each couple of rat sub-lines. # = significantly different, naïve/experienced RLA/NELR subline vs. naïve/experienced RHA/NEHR rat sub-line.

**Table 12:** Basal levels of extracellular dopamine in the dialysate from the nucleus accumbens and the medial prefrontal cortex of RHA/RLA rat sub-lines at the first (sexually naïve condition) and at the fifth copulatory test (sexually experienced condition) with a sexually receptive female rat.

Rat Sub-Lines	Sexual Condition	Nucleus Accumbens Extracellular Dopamine (nM)	Medial Prefrontal Cortex Extracellular Dopamine (nM)
RHA	Naive	1.9	0.8
	Experienced	2.0	1.2
RLA	Naive	1.9	0.7
	Experienced	1.8	1.1

Experimental details and data are in [254,255].

**Table 13:** Extracellular dopamine increases in the dialysate obtained from the nucleus accumbens and the medial prefrontal cortex of RHA and RLA rats during sexual activity at the first (sexually naïve condition) and at the fifth copulatory test (sexually experienced condition) with a receptive female rat.

Rat Sub-Lines	Sexual Condition	Area under the Curve of Extracellular Dopamine Values in the Nucleus Accumbens Dialysate	Area under the Curve of Extracellular Dopamine Values in the Medial Prefrontal Cortex dialysate
RHA	Naive	281	210
	Experienced	256	315 *
RLA	Naive	95 #	154 #
	Experienced	164 *	252 *

Experimental details and data are in [254,255]. * = significantly different, experienced vs. naive, same rat sub-line, for each couple of rat sub-lines. # = significantly different, naïve/experienced RLA subline vs. naïve/experienced RHA rat sub-line.

**Table 14:** Mean values of copulatory parameters of the first series of copulatory activity of male LY/HY rat sub-lines at the first copulatory test with a receptive female rat (sexually naïve condition) and after at least 5 copulatory tests (sexually experienced condition): comparison with genetically heterogeneous Sprague-Dawley (SD) rats.

	Psychogenetically Selected Rat Sub-Lines					
Copulatory Parameters	LY		HY		SD	
	Naive	Experienced	Naive	Experienced	Naive	Experienced
ML	683	93 *	694	140 *	400	25 *
IL	2590	187 *	1499	226 *	500	30 *
EL	2655	345 *	2700	520 *	1200	520 *
MF	na	17	na	13	15	6 *
IF	na	10	na	8	21	10 *
PEI	na	300 *	na	380 *	470	360
EF	na	na	na	na	1	2.7 *
III	na	70	na	120 #	60	30 *
CE	na	0.59	na	0.45 #	0.60	0.72 *

ML, IL, EL = mount/intromission/ejaculation latency (seconds); MF, IF, EF = mount/intromission/ejaculation frequency; PEI = postejaculatory interval (seconds); III = inter intromission interval (seconds); CE = copulatory efficiency (for definitions see legend of Table 8 and Table 11). Experimental details and data for HY/LY rats are in [280] and Sprague-Dawley rats in [248]. na = not available. * = significantly different, experienced vs. naive, same rat sub-line, for each couple of rat sub-lines. # = significantly different, naïve/experienced HY subline vs. naïve/experienced LY rat sub-line.

**Table 15:** Mean values of copulatory parameters of the first series of copulatory activity of male DAT-KO, HET and WT rat sub-lines at the first (sexually naïve condition) and at the fifth copulatory test (sexually experienced condition) with a receptive female rat.

	Rat Sub-Lines					
Copulatory Parameters	DAT-KO		HET		WT	
	Naive	Experienced	Naive	Experienced	Naive	Experienced
NCPE	0.5 #	0.5 #	1.8	3 * #	1	1.2
ML	180	20 *	200	20 *	560	50 *
IL	210	17 *	360	26 *	580	40 *
EL	980	445 *	1320	920	1420	1020 *
MF	10	7 *	20	20	14	13
IF	10	9	11	12	10	14
PEI	250 #	260 #	480	280 *	470	360
EF	2.2	3.5 *	1.3	2.7 *	1.1	2.4 *
III	350 #	30 *	360	40 *	700	50 *
CE	0.50 #	0.56 * #	0.46	0.48	0.41	0.52 *

NCPE = non-contact penile erections; ML, IL, EL = mount/intromission/ejaculation latency (seconds); MF, IF, EF = mount/intromission/ejaculation frequency; PEI = post ejaculatory interval (seconds); III = inter intromission interval (seconds); CE = copulatory efficiency (for definitions see legend of Table 8 and Table 11). Experimental details and data are in [296]. * = significantly different, experienced vs. naive, same rat sub-line, for each couple of rat sub-lines. # = significantly different, naïve/experienced DAT-KO/HET sub-lines vs. naïve/experienced WT rat sub-lines.

**Table 16:** Basal levels of extracellular dopamine in the nucleus accumbens shell of DAT-KO, HET and WT rat sub-lines at the first (sexually naïve condition) and at the fifth copulatory test (sexually experienced condition) with a receptive female rat.

Rat Sub-Lines	Sexual Condition	Extracellular Dopamine (nM)
WT	Naive	1.8
	Experienced	2.0
HET	Naive	4.5 *
	Experienced	5.3 *
DAT-KO	Naive	17 * #
	Experienced	19 * #

Experimental details and data are in [296]. * = significantly different, naïve/experienced DAT-KO and HET vs. naïve/experienced WT. # = significantly different, naïve/experienced DAT-KO vs. naïve/experienced HET.

**Table 17:** Extracellular dopamine increases in the nucleus accumbens shell of WT, HET and DAT-KO rats during sexual activity in the fifth copulatory test with a receptive female rat.

Rat Sub-Lines	Area under the Curve of Extracellular Dopamine Values
WT	300
HET	680 *
DAT-KO	2200 * #

Experimental details and areas under the curve were obtained by the data found in [296]. * = significantly different, DAT-KO and HET vs. WT. # = significantly different, DAT-KO vs. HET.
